# MiR‐17‐5p‐engineered sEVs Encapsulated in GelMA Hydrogel Facilitated Diabetic Wound Healing by Targeting PTEN and p21

**DOI:** 10.1002/advs.202307761

**Published:** 2024-01-29

**Authors:** Qian Wei, Jianlong Su, Sheng Meng, Yaxi Wang, Kui Ma, Bingmin Li, Ziqiang Chu, Qilin Huang, Wenzhi Hu, Zihao Wang, Lige Tian, Xi Liu, Tanshi Li, Xiaobing Fu, Cuiping Zhang

**Affiliations:** ^1^ Research Center for Tissue Repair and Regeneration Affiliated to the Medical Innovation Research Division Chinese PLA General Hospital Beijing 100048 P. R. China; ^2^ Research Unit of Trauma Care Tissue Repair and Regeneration Chinese Academy of Medical Sciences 2019RU051 Beijing 100048 P. R. China; ^3^ Chinese PLA Medical School Beijing 100853 P. R. China; ^4^ Department of Emergency The First Medical Center Chinese PLA General Hospital Beijing 100853 P. R. China; ^5^ PLA Key Laboratory of Tissue Repair and Regenerative Medicine and Beijing Key Research Laboratory of Skin Injury, Repair and Regeneration Beijing 100048 P. R. China; ^6^ Innovation Center for Wound Repair West China Hospital Sichuan University Chengdu Sichuan 610041 P. R. China

**Keywords:** miR‐17‐5p, angiogenesis, collagen deposition, diabetic wounds, small extracellular vesicles

## Abstract

Delayed wound healing is a major complication of diabetes, and is associated with impaired cellular functions. Current treatments are unsatisfactory. Based on the previous reports on microRNA expression in small extracellular vesicles (sEVs), miR‐17‐5p‐engineered sEVs (sEVs^17‐OE^) and encapsulated them in gelatin methacryloyl (GelMA) hydrogel for diabetic wounds treatment are fabricated. SEVs^17‐OE^ are successfully fabricated with a 16‐fold increase in miR‐17‐5p expression. SEVs^17‐OE^ inhibited senescence and promoted the proliferation, migration, and tube formation of high glucose‐induced human umbilical vein endothelial cells (HG‐HUVECs). Additionally, sEVs^17‐OE^ also performs a promotive effect on high glucose‐induced human dermal fibroblasts (HG‐HDFs). Mechanism analysis showed the expressions of p21 and phosphatase and tensin homolog (PTEN), as the target genes of miR‐17‐5p, are downregulated significantly by sEVs^17‐OE^. Accordingly, the downstream genes and pathways of p21 and PTEN, are activated. Next, sEVs^17‐OE^ are loaded in GelMA hydrogel to fabricate a novel bioactive wound dressing and to evaluate their effects on diabetic wound healing. Gel‐sEVs^17‐OE^ effectively accelerated wound healing by promoting angiogenesis and collagen deposition. The cellular mechanism may be associated with local cell proliferation. Therefore, a novel bioactive wound dressing by loading sEVs^17‐OE^ in GelMA hydrogel, offering an option for chronic wound management is successfully fabricated.

## Graphical abstract

miR‐17‐5p‐engineered sEVs (sEVs^17‐OE^) can restore cellular functions of skin repair cells in diabetic wounds by inhibiting senescence and promoting angiogenesis and collagen deposition. Furthermore, a functional wound dressing by loading sEVs^17‐OE^ into GelMA hydrogel is developed, accelerating the diabetic wound healing process and offering an option for chronic wound management.

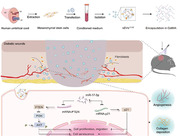

## 1 Introduction

With the aging population and increasing obesity rates, the prevalence of chronic refractory wounds, including diabetic foot ulcers (DFU), has increased significantly. The International Diabetes Federation (IDF) reports that every 20 s, a diabetic patient is hospitalized for a lower limb amputation worldwide.^[^ ^1^, ^2^ ^]^ Pathologically, insufficient vascularization and the reduced proliferation and migration of skin repair cells, such as endothelial cells and fibroblasts are the primary causes of delayed or impaired wound healing,^[^ ^3^, ^4^ ^]^ which may result from cellular senescence induced by a hyperglycemic environment.^[^ ^5^, ^6^, ^7^ ^]^ Current treatments for diabetic wounds, such as debridement and skin flap transfer, have proven unsatisfactory, underscoring the need for more efficient diabetic wound management.

Small extracellular vesicles (sEVs) or exosomes derived from various stem cells have garnered attention for their therapeutic potential. SEVs are 50–150 nm‐sized membrane‐enclosed vesicles. SEVs encapsulate and deliver functional molecules such as microRNAs (miRNAs) and proteins, to recipient cells for cell‐cell communication. SEVs offer advantages over donor cells, including higher stability under physiological conditions, fewer ethical concerns, a reduced risk of immune response, and a low risk of embolism and carcinogenicity.^[^ ^8^ ^]^ They can protect and deliver functional molecules such as miRNAs and proteins, to recipient cells. At present, mesenchymal stem cells (MSCs) are widely used as donor cells for sEV isolation. Moreover, MSC‐sEVs have proved to be promising substitutes for MSCs with equal or better therapeutic effects as well.^[^ ^9^ ^]^ However, the therapeutic outcomes of natural MSC‐sEVs are often limited by insufficient therapeutic cargo. To this end, engineering sEVs for efficient loading of therapeutic molecules may provide a potential solution.

MiRNAs, as part of the exosomal cargo, can repress the target gene expression via binding the 3′ untranslated regions of mRNA post‐transcriptionally.^[^ ^10^ ^]^ Due to the inherent susceptibility of miRNAs to degradation, effective delivery systems are required to transfer miRNAs to target cells. Reportedly, miRNA is stable in sEVs and sEVs protect them in an enzyme‐rich environment. Free miRNAs dramatically degraded just after 1 to 24 h,^[^ ^11^, ^12^, ^13^ ^]^; on the contrary, miRNA encapsulated in the sEVs remained stable and the level was more than 90% of the origin level after 48 h, displaying the promising vehicles for miRNA delivery.^[^ ^13^ ^]^ MiR‐17‐5p has been recognized as a crucial mediator of many diseases through the optimization of cellular functions.^[^ ^14^, ^15^, ^16^ ^]^ For example, miR‐17‐5p was found to have therapeutic effects for brain tissue damage,^[^ ^17^ ^]^ and osteoarthritis.^[^ ^18^ ^]^ Furthermore, overexpression of miR‐17‐5p was found to protect from high glucose (HG)‐induced endothelial cell injury.^[^ ^19^ ^]^ We have recently found that sEVs derived from human umbilical cord MSCs (hucMSCs) could accelerate diabetic wound healing and miR‐17‐5p was found to be enriched in hucMSC‐sEVs.^[^ ^3^ ^]^ MiR‐17‐5p was a crucial regulator by enhancing the angiogenic effect of endothelial cells,^[^ ^3^ ^]^ and exerting protective effects on fibroblasts in vitro and in vivo.^[^ ^20^ ^]^ This suggests that engineered sEVs modified for the efficient loading of miR‐17‐5p may provide a promising and novel therapy for diabetic wounds.

Gelatin methacryloyl (GelMA) hydrogel is a kind of photo‐crosslinked biological hydrogel with tailored morphologies, manageable mechanical and degradation properties, high transparency, good biocompatibility, high moisturizing effect, and efficient barrier properties.^[^ ^21^, ^22^ ^]^ Meanwhile, as the backbone of GelMA, gelatin offers unique features such as proteolytic degradability, and suitable cell adhesion sites and is well‐sourced.^[^ ^23^ ^]^ Besides, we have encapsulated VH298‐loaded sEVs^[^ ^24^ ^]^ or autophagosomes,^[^ ^25^ ^]^ into porous GelMA hydrogel to prolong their retention time to 15 days and 8 days, respectively, and thereby accelerate the diabetic wound healing process. Thus, GelMA hydrogels are ideal carriers for engineered sEVs.

In this study, we fabricated miR‐17‐5p‐engineered sEVs (sEVs^17‐OE^) and encapsulated them in a gelatin methacryloyl (GelMA) hydrogel for the treatment of diabetic wounds. SEVs^17‐OE^ were isolated from hucMSCs transfected with a lentivirus carrying miR‐17‐5p. After characterization, sEVs^17‐OE^ were added to a medium containing HG‐induced human umbilical vein endothelial cells (HG‐HUVECs) and human dermal fibroblasts (HG‐HDFs). The effects of sEVs^17‐OE^ on the functions of HG‐HUVECs and HG‐HDFs were observed and the mechanism responsible for the effects was explored. Finally, we loaded sEVs^17‐OE^ in GelMA hydrogel to fabricate a novel bioactive wound dressing for wound treatment. The results showed that in vitro, sEVs^17‐OE^ optimized the functions of HG‐HUVECs and HG‐HDFs by targeting p21 and phosphatase and tensin homolog (PTEN). In vivo, Gel‐sEVs^17‐OE^ effectively facilitated diabetic wound healing by enhancing local angiogenesis and collagen deposition.

## 2 Results

### 2.1 Fabrication and Characterization of miR‐17‐5p‐Engineered Small Extracellular Vesicles (sEVs^17‐OE^)

**Figure 1:**
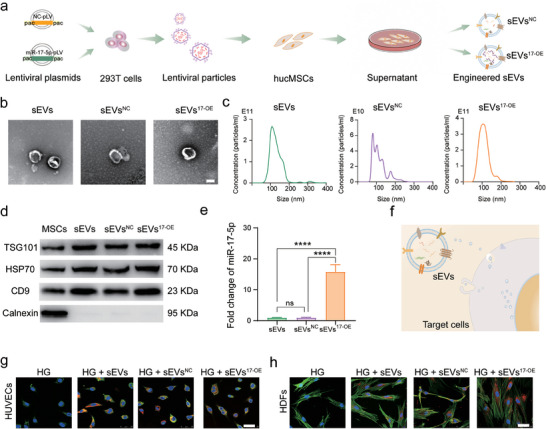
Fabrication and characterization of miR‐17‐5p‐engineered sEVs^17‐OE^. a) Schematic diagram illustrating the preparation process of sEVs, sEVs^NC^, and sEVs^17‐OE^. b) Typical TEM images of sEVs, sEVs^NC,^ and sEVs^17‐OE^ (scale bar = 100 nm). c) Particle size distribution of sEVs, sEVs^NC,^ and sEVs^17‐OE^ detected by NTA. d) Western blot analysis showing protein expressions of TSG101, HSP70, CD9 and Calnexin in MSCs, sEVs, sEVs^NC^ and sEVs^17‐OE^. e) qRT‐PCR analysis showing the relative expression level of miR‐17‐5p in sEVs, sEVs^NC^ and sEVs^17‐OE^ (*n* = 3 per group). f) Schematic illustration of sEVs internalization by the target cells. g,h) Respective internalization of sEVs, sEVs^NC,^ and sEVs^17‐OE^ by HG‐HUVECs (g) and HG‐HDFs (h) (scale bar = 50 µm). The data are presented as the mean ± SD. Differences among the groups were examined with one‐way ANOVA via Tukey's posttest. ^****^ *p* < 0.0001, ns, not significant versus the indicated group.

The hucMSCs were transfected with Lv‐miR‐17‐5p or Lv‐NC (Figure S1, Supporting Information). sEVs^17‐OE^ and sEVs^NC^ were isolated from the engineered hucMSCs (**Figure** **1**a). Natural sEVs isolated from the unmodified hucMSCs were used as negative controls. Transmission electron microscope (TEM), nanoparticle tracking analysis (NTA), and western blot analysis were employed to characterize the morphology, size distribution, and biomarkers of the three types of sEVs, respectively. TEM revealed a characteristic saucer‐like ultrastructure of these sEVs (Figure 1b). NTA results demonstrated that there were no significant differences in size distribution or mean diameter among these sEVs (Figure 1c). The mean diameters of sEVs, sEVs^NC^, and sEVs^17‐OE^ were ≈130.3 nm, 111.4 nm, and 124.0 nm, respectively. The particle concentrations of the three groups were 8.46 × 10^10^ particles mL^−1^ for sEVs, 7.96 × 10^10^ particles mL^−1^ for sEVs^NC^, and 1.04 × 10^11^ particles mL^−1^ for sEVs^17‐OE^, separately harvested from ≈5 × 10^8^ cells. Representative surface markers, including TSG101, HSP70, and CD9, were highly expressed in all sEVs, whereas the endoplasmic reticulum (ER) marker Calnexin was detected only in MSCs (Figure 1d), excluding cellular contamination during the isolation of sEVs. Then, qRT‐PCR was used to investigate the levels of miR‐17‐5p in sEVs, sEVs^17‐OE^, and sEVs^NC^ (Figure 1e) as well. Obviously, the miR‐17‐5p level was increased by ≈16 times in sEVs^17‐OE^ compared to that in sEVs or sEVs^NC^, indicating successful engineering of sEVs with miR‐17‐5p (Figure 1e; *p* < 0.0001). Hence, sEVs^17‐OE^ were successfully fabricated and isolated.

Successful internalization of sEVs by target cells is the prerequisite to performing functional modulation (Figure 1f). Upon reaching recipient cells, sEVs are internalized through possible ways including direct fusion, receptor‐mediated endocytosis, phagocytosis, and receptor interaction.^[^ ^26^, ^27^ ^]^ SEVs were labeled with DiI (red fluorescence) and coincubated with HG‐HUVECs or HG‐HDFs for 5–6 h. Red fluorescence of sEVs appeared around the nucleus of HG‐HUVECs or HG‐HDFs, implying that these three sEVs could be favorably internalized into perinuclear regions of cells (Figure 1g,h) via the above‐mentioned means as expected. Then, sEVs were surmised to fuse with the endoplasmic reticulum, mitochondria, and trans‐Golgi to release cargos, including miR‐17‐5p, into the target cells.^[^ ^26^ ^]^ Nonetheless, the detailed mechanisms underlying various pathways by which sEVs release cargo are further investigated.

### 2.2 SEVs^17‐OE^ Optimized Endothelial Cell Function by Targeting p21 and Phosphatase and Tensin Homolog (PTEN)

**Figure 2:**
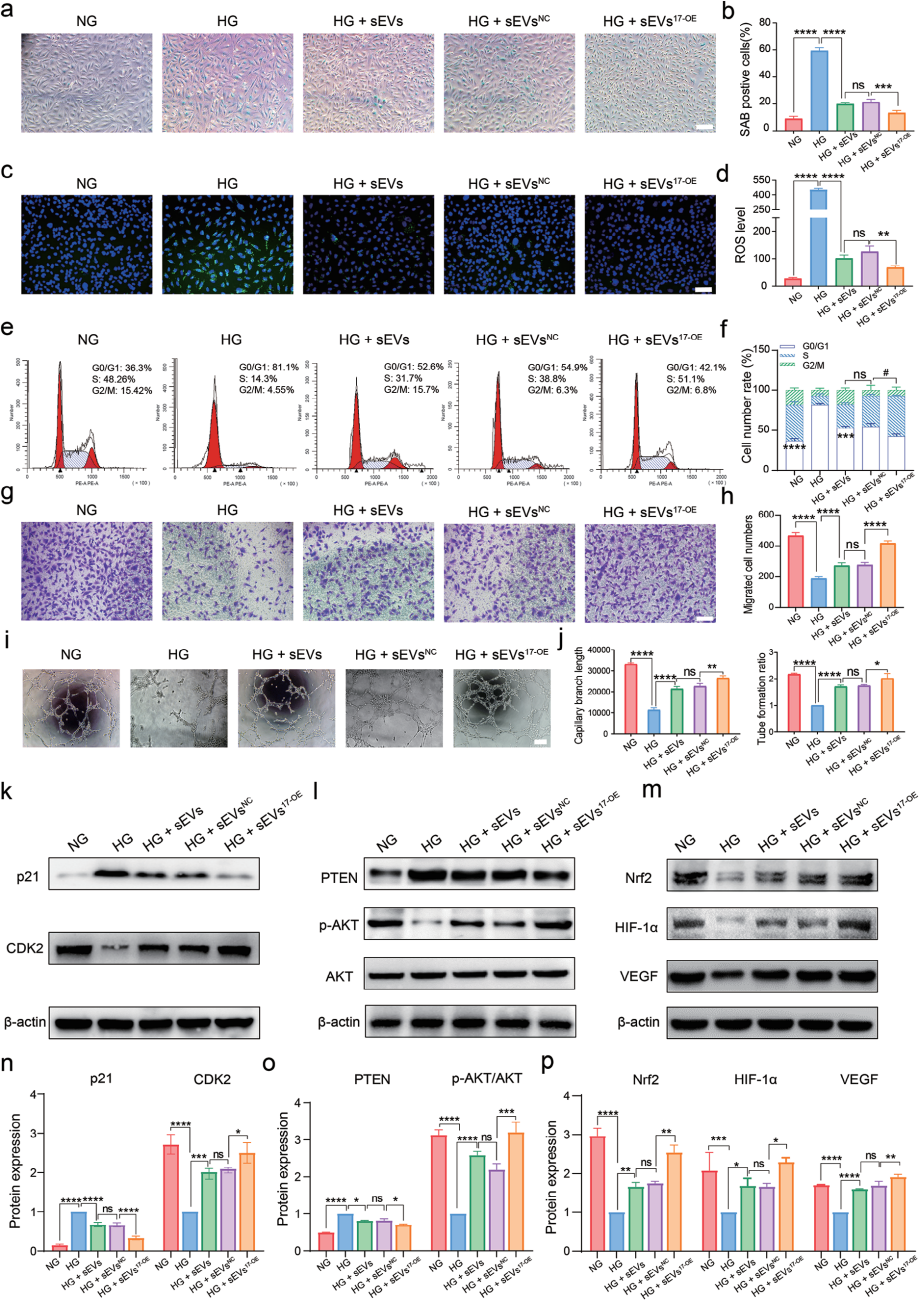
SEVs^17‐OE^ optimized endothelial cell function by targeting p21 and PTEN. a,b) SAB showing senescence in HUVECs treated with different groups. (*n* = 3 per group, scale bar = 100 µm). c,d) ROS level in HUVECs treated with different groups estimated by DCFH‐DA (*n* = 3 per group, scale bar = 100 µm). e,f) Flow cytometry revealing cell cycle and cell subgroups of HUVECs treated with different groups (*n* = 3 per group). g,h) Transwell assay representing migration properties of HUVECs with the above treatments (*n* = 3 per group, scale bar = 100 µm). i,j) Representative images of in vitro angiogenesis assay (scale bar = 100 µm), quantification results of capillary branch length, and tube formation ratio in HUVECs treated with different groups (*n* = 3 per group). k–p) Western blot analysis (k‐m) of the protein expression and corresponding quantification results (n‐p) of p21, CDK2, PTEN, p‐AKT, AKT, Nrf2, HIF‐1α, VEGF inside HUVECs treated with NG, HG, HG + sEVs, HG +sEVs^NC,^ and HG +sEVs^17‐OE^. Protein expression levels were normalized to β‑actin levels (*n* = 3 per group). The data are presented as the mean ± SD. Differences among the groups were examined with one‐way ANOVA with Tukey's posttest. ^*^ *p* < 0.05, ^**^ *p* < 0.01, ^***^ *p* < 0.001, ^****^ *p* < 0.0001, ns, not significant versus the indicated group.

Reportedly, β‐Galactosidase staining (SAB) was highly expressed in senescent HG‐HUVECs.^[^ ^28^ ^]^ The number of SAB‐positive cells (dyed blue) was significantly decreased upon treatment with all three sEVs, with the sEVs^17‐OE^ group showing a lower number of positive cells (**Figure** **2**a,b). Admittedly, excessive intracellular ROS generation causes cell senescence in the diabetic microenvironment.^[^ ^31^ ^]^ Here, we examined whether sEVs^17‐OE^ scavenged intercellular ROS. Administration of sEVs^17‐OE^ reduced ROS production to the greatest extent, whereas administration of sEVs^NC^ or natural sEVs alleviated ROS levels to a lesser extent, as demonstrated by the green fluorescence in Figure 2c,d. Additionally, we also investigated the anti‐senescence effect of sEVs^17‐OE^ on HG‐HUVECs by measuring the expression levels of senescence‐associated proteins such as p53 and p16. As expected, p53 and p16 expression was moderately downregulated in HG‐HVUECs treated with sEVs or sEVs^NC^ and reduced heavily with sEVs^17‐OE^ (Figure S2, Supporting Information).

Next, the proliferative abilities of HG‐HUVECs upon application of different treatments we tested by conducting cell cycle and EdU assays. Flow cytometry was employed to estimate the proportions of the three cellular subpopulations (G0/G1, S, and G2/M) based on their DNA content.^[^ ^29^ ^]^ Generally, higher proportions of S and G2/M cells represent a stronger proliferative ability. HG was found to inhibit the G1‐S transition of HUVECs, whereas co‐incubation with sEVs, sEVs^NC^, or sEVs^17‐OE^ reversed cell cycle arrest. Furthermore, the proportions of S and G2/M cells were higher in the sEV^17‐OE^ group than those in the sEV and sEV^NC^ groups (Figure 2e,f). In addition, a higher ratio of Edu‐positive cells (green fluorescence) was observed in the sEVs, sEVs^NC^, and sEVs^17‐OE^ groups with the ratio being the highest in the sEVs^17‐OE^ group (Figure S3, Supporting Information). Consistently, treatment with sEVs or sEVs^17‐OE^ significantly improved the viability of HG‐HUVECs, and the enrichment of miR‐17‐5p further boosted these improvements. Additionally, we identified a dose‐dependent effect of miR‐17‐5p in sEVs on the proliferative ability of HG‐HUVECs using the cell counting kit‐8 (CCK‐8) assay (Figure S4a, Supporting Information).

Transwell and scratch assays were performed to assess the effect of sEVs^17‐OE^ on HG‐HUVEC migration in vitro. Figure 2g,h showed that all types of sEVs significantly elevated the number of migrated cells (purple‐stained) compared to the HG group with the number of migrated cells being the highest in the sEV^17‐OE^ group. Similarly, in the scratch gap closure assay, sEVs^17‐OE^, sEVs^NC,^ and natural sEVs induced a significant increase in gap closure in HG‐HUVECs compared with the PBS control (Figure S5a,b, Supporting Information). Additionally, the sEVs^17‐OE^‐treated group acquired a larger migratory area than the sEVs^NC^ or sEVs‐treated groups. Collectively, miR‐17‐5p‐enriched sEVs^17‐OE^ enhanced the migratory ability of HG‐HUVECs. Next, we investigated the effect of sEVs^17‐OE^ on the angiogenic properties of HG‐HUVECs. Figure 2i,j shows that the best tube formation performance was observed in sEVs^17‐OE^‐treated HG‐HUVECs, which showed the highest capillary branches and a complete tubular structure. Therefore, sEVs boosted the angiogenic ability of HG‐HUVECs, with sEV^17‐OE^ group functioning to the greatest extent.

To explore the mechanisms underlying the optimized phenotypes of HG‐HUVECs following sEVs^17‐OE^ treatment, we examined the expression levels of target genes of miR‐17‐5p. Reportedly, p21 is one of the potential target genes of miR‐17‐5p, accounting for the G1 checkpoint of the cell cycle together with CDK2.^[^ ^30^ ^]^ Western blot analysis verified the enhanced p21 levels and reduced CDK2 levels inside HG‐HVUECs, consistent with the previous report^[^ ^30^ ^]^ and associated with cellular senescence. p21 expression was moderately downregulated by treating HG‐HVUECs with sEVs or sEVs^NC^ and reduced heavily with sEVs^17‐OE^; whereas the opposite tendency was observed in the expression of CKD2. CDK2 expression was downregulated in HG‐HUVECs. Specifically, its expression was partially reversed after sEV or sEV^NC^ administration and recovered significantly after sEV^17‐OE^ administration (Figure 2k,n).

We previously reported that miR‐17‐5p in sEVs significantly repressed the expression of PTEN, thus stimulating the p‐AKT/AKT pathway.^[^ ^3^ ^]^ Here, we reasoned that miR‐17‐5p enriched hucMSC‐sEVs^17‐OE^ would exert a more protective effect than natural hucMSC‐sEVs. Indeed, the western blot indicated that HG‐HUVECs treated with sEVs^17‐OE^ displayed a lower level of PTEN than those treated with sEVs or sEVs^NC^. Upon inhibition of PTEN expression, the expression level of p‐AKT/AKT increased gradually with the treatment with sEVs, sEVs^NC^, and sEVs^17‐OE^ (Figure 2l,o). According to literature, inhibiting PTEN could not only boost cell growth and migration but also increase the expression of nuclear factor erythroid2‐related factor 2 (Nrf2) to combat excess ROS, thus rescuing ROS‐dependent senescence in several pathological conditions.^[^ ^31^ ^]^ Therefore, we measured Nrf2 protein levels and functional proteins in HUVECs as well. Figure 2m–p showed that the expression level of Nrf2 was recovered moderately by applying sEVs, sEVs^NC^, and restored significantly by applying sEVs^17‐OE^. Similar results were observed in HIF‐1α and VEGF expression. Consequently, the activated p‐AKT/AKT pathway was considered to upregulate the expression levels of Nrf2 to counter excessive ROS, and those of HIF‐1α and VEGF to boost angiogenesis.

### 2.3 SEVs^17‐OE^ Optimized the Function of Fibroblasts by Targeting p21 and PTEN

**Figure 3:**
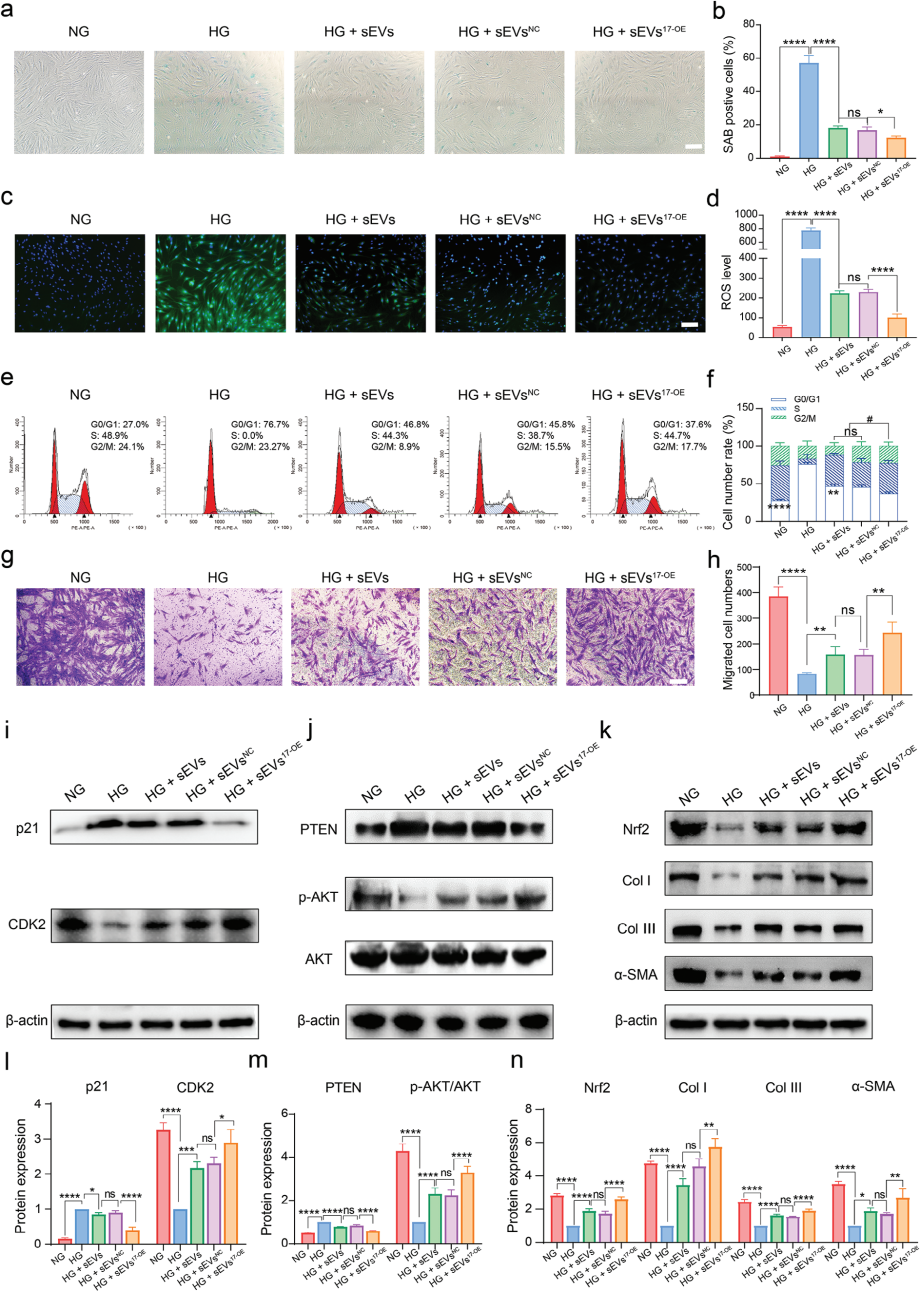
SEVs^17‐OE^ optimized the function of fibroblasts by targeting p21 and PTEN. a,b) SAB shows the senescence in HDFs with the above‐mentioned treatments (*n* = 3 per group, scale bar = 100 µm). c,d) ROS level in HDFs treated with different groups (*n* = 3 per group, scale bar = 100 µm). e,f) Flow cytometry detecting cell cycle in HDFs with the above‐mentioned treatments (*n* = 3 per group). g,h) Migration abilities of HDFs after relevant treatments detected by transwell assay (*n* = 3 per group, scale bar = 100 µm). i–n) Western blot analysis showing p21/CDK2 and PTEN/p‐AKT/Nrf2/Col I/Col III/α‐SMA signaling pathways in HDFs treated with NG, HG, HG + sEVs, HG + sEVs^NC,^ and HG + sEVs^17‐OE^. Protein expression levels were normalized to β‑actin levels (*n* = 3 per group). The data are presented as the mean ± SD. Differences among the groups were examined with one‐way ANOVA with Tukey's posttest. ^*^ *p* < 0.05, ^**^ *p* < 0.01, ^***^ *p* < 0.001, ^****^ *p* < 0.0001, ns, not significant versus the indicated group.

Furthermore, we investigated the effect of sEVs^17‐OE^ on mitigating senescence and promoting proliferation and migration abilities of HG‐HDFs, which is another important participant during the regeneration process. We also found that the number of SAB positive HDFs (blue) was much larger when treated with HG compared to NG. The number of SAB‐positive cells diminished significantly after treatment with all three sEVs with the sEV^17‐OE^ group showing the lowest number of cells (**Figure** **3**a,b). Next, we examined ROS levels inside HG‐HDFs following different treatments. Apparently, the HG microenvironment was found to facilitate ROS accumulation compared with the NG microenvironment, as demonstrated by the increased green fluorescence signal in Figure 3c. However, the administration of sEVs^17‐OE^ significantly repressed ROS production inside HG‐HDFs, compared to that of the administration of sEVs^NC^ or sEVs (Figure 3c,d). Additionally, we also detected p53 and p16 levels, which were moderately downregulated by treating HG‐HDFs with sEVs or sEVs^NC^ and heavily reduced by sEVs^17‐OE^ (Figure S6, Supporting Information).

Subsequently, the viability of HDFs with different treatments was examined via cell cycle and Edu assays. For the cell cycle test, HG inhibited the G1‐S transition of HDFs, whereas the administration of sEVs, sEVs^NC^, or sEVs^17‐OE^ rescued the cell cycle arrest, with the ratio of cells in the S and G2/M phases being higher in the sEV^17‐OE^ group than in the sEV or sEV^NC^ group (Figure 3e,f). Additionally, a higher ratio of Edu‐positive cells was observed in the sEVs, sEVs^NC^, and sEVs^17‐OE^ treated groups, with the highest ratio being observed in the sEV^17‐OE^ group (Figure S7, Supporting Information). Consistently, treatment with sEVs or sEVs^17‐OE^ significantly improved the proliferation of HG‐HDFs, and miR‐17‐5p overexpression further enhanced these effects. Meanwhile, we have verified the dose‐dependent effect of miR‐17‐5p in sEVs on proliferative ability of HG‐HDFs using CCK‐8 assay that cell viability of HG‐HDFs was increased gradually with the increasing concentration of sEVs^17‐OE^ from 1 × 10^10^ to 2 × 10^10^ particles mL^−1^ (Figure S4b, Supporting Information). Transwell and scratch assays were employed to assess the effect of sEVs^17‐OE^ on the migratory ability of HG‐HDFs. Figure 3g,h showed that all types of sEVs significantly increased the number of migrated cells compared to that of the control group. Furthermore, the number of migrated cells was the largest in sEVs^17‐OE^‐treated group. Similarly, in the scratch gap assay, sEVs^17‐OE^, sEVs^NC^ and natural sEVs stimulated a markable increase in gap closure compared to that in the control group (Figure S8, Supporting Information). SEVs^17‐OE^ treated group achieved a larger migratory area than the sEV^NC^ and sEV groups. Therefore, sEVs boosted the migratory ability of HDFs, and miR‐17‐5p‐enrichment exhibited higher efficacy.

Western blot analysis confirmed enhanced p21 and reduced CDK2 levels in HG‐HDFs. In contrast, treatment of HG‐HDFs with sEVs or sEVs^NC^ significantly downregulated p21 and upregulated CDK2 expression, respectively. The most significant changes were observed in the sEV^17‐OE^ group (Figure 3i,l). Meanwhile, the western blot showed that HG‐HDFs treated with sEVs^17‐OE^ had lower levels of PTEN than those treated with sEVs or sEVs^NC^. Additionally, the administration of sEVs, sEVs^NC,^ and sEVs^17‐OE^ increased the levels of p‐AKT/AKT in HG‐HGFs. Administration of sEVs, sEVs^NC,^ and sEVs^17‐OE^ increased the levels of p‐AKT/AKT in HG‐HGFs (Figure 3j,m). Consequently, the Nrf2 level was increased in the sEV, sEV^NC,^ and sEV^17‐OE^ groups, displaying a robust ROS scavenging response; a similar trend was observed in the expressions of Col I, Col III, and α‐SMA in the above groups, indicating enhanced collagen‐secretion abilities (Figure 3k,n). Notably, these proteins were more abundant in the sEVs^17‐OE^ treated HG‐HDFs than those in the sEVs or sEVs^NC^ treated groups.

### 2.4 Fabrication and Characterization of sEV^17‐OE^‐loaded GelMA Hydrogel

**Figure 4:**
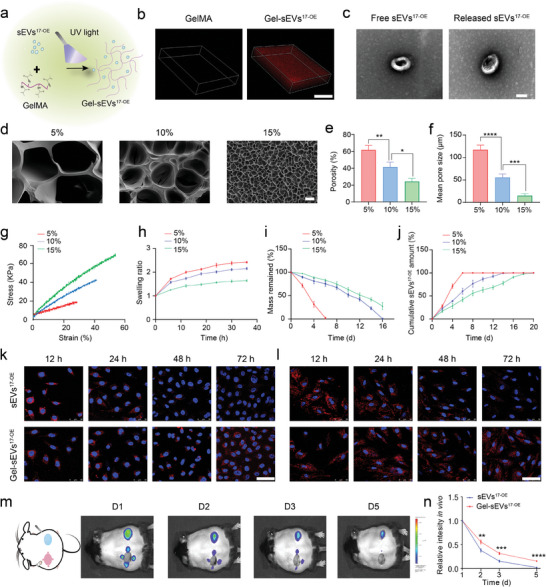
Fabrication and characterization of sEV^17‐OE^‐loaded GelMA hydrogel (Gel‐sEVs^17‐OE^). a) Schematic diagram depicting the preparation process of Gel‐sEVs^17‐OE^. b) CLSM images revealing the distribution of sEVs^17‐OE^ in GelMA hydrogel. c) Typical TEM images of free sEVs^17‐OE^ and sEVs^17‐OE^ released from GelMA hydrogel (scale bar = 100 nm). d) Representative cross‐section SEM images of GelMA hydrogels with different concentrations of 5%, 10%, and 15% (scale bar = 20 µm). e–i) Porosity, mean pore size, stress‐strain curves, swelling ratio, and degradation of GelMA hydrogels with concentrations of 5%, 10%, and 15%, respectively (*n* = 3 per group). j) Cumulative release profile of sEVs^17‐OE^ from GelMA hydrogel with different concentrations within 20 days (*n* = 3 per group). k,l) Typical fluorescent images of HG‐HUVECs and HG‐HDFs that were co‐incubated either with equivalent DiI‐stained sEVs^17‐OE^ (red) in free form or released from GelMA hydrogel, respectively, with nuclei labeled by Hoechst (blue). Scale bar = 50 µm. m) Typical in vivo imaging pictures reflecting retention of DiI‐stained sEVs^17‐OE^ in free form and GelMA encapsulation form on wounds at the designed time point. n) Quantitative results of released sEVs^17‐OE^ intensity in vivo of (m) (*n* = 3 per group). The data are presented as the mean ± SD. Differences among the groups were examined with one‐way ANOVA with Tukey's posttest. ^*^ *p* < 0.05, ^**^ *p* < 0.01, ^***^ *p* < 0.001, ^****^ *p* < 0.0001, ns, not significant versus the indicated group.

We loaded sEVs^17‐OE^ into a biocompatible GelMA hydrogel under UV‐photopolymerization to prolong their retention‐time at the wound sites (**Figure** **4**a). Confocal laser scanning microscopy (CLSM) images in Figure 4b indicated successful loading and uniform distribution of sEVs^17‐OE^ (red) within the GelMA hydrogel. Furthermore, sEVs^17‐OE^ released from GelMA hydrogel on day 3 remained morphologically intact with no visible differences compared to the free sEVs^17‐OE^, further highlighting their potential application in wound repair (Figure 4c). To optimize the retention‐time of sEVs^17‐OE^ and the swelling ratio of GelMA hydrogel, three types of GelMA hydrogel with different monomer concentrations were employed to load sEVs^17‐OE^. Figure 4d–f shows that increasing the monomer concentration from 5% to 15% led to a decrease in the porosity of GelMA hydrogel from 61.77% to ≈24.27%, and a significant reduction in pore size from 117.4 µm to 15.13 µm. Additionally, increasing the monomer concentration to 15% elevated the tensile breaking strength to 67.95 kPa and reduced the swelling ratio to ≈165% (Figure 4g,h). These were attributed to an increase in the crosslinking degree of GelMA hydrogel at high monomer concentrations. A low swelling ratio also reduced the mechanical force on the wound and prevented scar formation.^[^ ^32^ ^]^ As for degradation, GelMA hydrogel at 15% was degraded by ≈72%, while the one at 5% was degraded almost completely in PBS buffers (pH 7.2–7.4) on day 6 (Figure 4i). The lower porosity and degradation ratio prolonged the release time of sEVs^17‐OE^ from GelMA hydrogel (15%) to 20 days, which was beneficial for enhanced local delivery at wounds. The GelMA hydrogel (5%) released sEVs rapidly in the first few days (6 days), whereas the 15% group released sEVs in a more constant and sustained manner than the other two groups (Figure 4j). Therefore, GelMA hydrogel with a 15% monomer concentration was selected for the subsequent experiments. Additionally, good adhesiveness of the hydrogel to the tissue is also important for wound closure, as it offers a closed protective barrier to prevent infection and bleeding.^[^ ^27^ ^]^ The Gel‐sEVs^17‐OE^ hydrogel adhered well to pig skin after stretching, twisting, bending, and water washing (Figure S9a, Supporting Information), indicating its efficient adhesiveness in humid and dynamic environments. Furthermore, two pieces of 20% gelatin‐coated glass slides with their overlapping region (2 × 2 cm) bonded together with the Gel‐sEVs^17‐OE^ could withstand a weight of 100 g both in out‐of‐plane and in‐plane directions (Figure S9b,c, Supporting Information).

Next, the release of sEVs^17‐OE^ from GelMA was investigated in vitro (HG‐HUVECs and HG‐HDFs) and in vivo (skin wound on diabetic mice). In vitro, the release of sEVs^17‐OE^ and cellular internalization assay confirmed their sustained‐release profiles from GelMA hydrogel (Figure 4k). Specifically, free sEVs^17‐OE^ were rapidly internalized by recipient cells and then degraded after 72 h, whereas the release of sEVs^17‐OE^ from GelMA hydrogel was continuous and the red fluorescence signal of sEVs^17‐OE^ remained stable even at 72 h in the HG‐HUVECs (Figure 4k) and HG‐HDFs (Figure 4l). Next, two full‐thickness wounds (diameter of ≈1 cm) were created on both sides of a diabetic mouse. One wound was treated with sEV^17‐OE^‐loaded GelMA hydrogel, whereas the other one was treated with equivalent free sEVs^17‐OE^ by four‐point injection (Figure 4m). Figure 4n showed that free sEVs^17‐OE^ spread quickly around the wound and were metabolized almost completely on day 5 postinjection. In contrast, the fluorescence of sEVs^17‐OE^ released from GelMA hydrogel was detected obviously on day 5, indicating a sustained release of sEVs^17‐OE^. Therefore, GelMA not only preserved the intactness of sEVs^17‐OE^, but also achieved a sustained release of sEVs^17‐OE^. Furthermore, sEV^17‐OE^‐loaded GelMA hydrogel and GelMA hydrogel were found to be highly biocompatible. The live/dead staining assay showed no significant differences in the viability of HG‐HUVECs and HG‐HDFs cultured with prepolymer solution of sEV^17‐OE^‐loaded GelMA, GelMA, and blank control groups (Figure S10, Supporting Information). In addition, H&E staining of the heart, lung, liver, kidney, and spleen of the mice treated with sEVs^17‐OE^‐loaded GelMA hydrogel demonstrated no histological abnormalities or immune cell infiltration in comparison with those treated with GelMA hydrogel or PBS (Figure S11, Supporting Information). Hence, these results indicate that sEVs^17‐OE^‐loaded GelMA hydrogels are highly biocompatible, both in vitro and in vivo. Furthermore, we have also examined the effects of Gel‐sEVs^17‐OE^ on NG‐HUVECs and NG‐HDFs briefly and observed the positive regenerative effects. Gel‐sEVs^17‐OE^ could promote the abilities of proliferation, migration, and tube formation of NG‐HUVECs (Figure S12, Supporting Information) and enhance the abilities of proliferation and migration of NG‐HDFs (Figure S13, Supporting Information) as well.

### 2.5 SEV^17‐OE^‐Loaded GelMA Hydrogel (Gel‐sEVs^17‐OE^) Accelerated Diabetic Wound Healing

**Figure 5:**
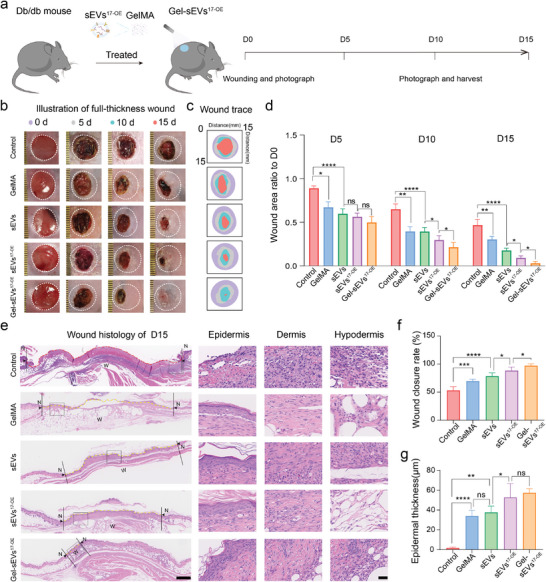
SEV^17‐OE^‐loaded GelMA hydrogel accelerated diabetic wound healing in vivo. a) Schematic diagram demonstrating the experimental procedure of full‐thickness excisional wound healing study on diabetic mice. b) Representative macroscopic images of wound healing in mice treated with control (PBS), GelMA hydrogel, sEVs, sEVs^17‐OE,^ and Gel‐sEVs^17‐OE^. c) Healing trace of wounds treated with different groups. d) Quantification of respective wound areas on day 5, day 10, and day 15 in mice with the above‐mentioned treatments in relation to their corresponding initial size on day 0 (*n* = 6 per group). e) H&E staining evaluation of wound regeneration with relevant treatments on day 15 showing morphometric analysis of newly formed epithelium (yellow line) and wound width (between two black arrows), scale bar: 1 mm and 100 µm for enlarged images. f,g) Statistics of wound closure rate and epidermis thickness in wounds with the above‐mentioned treatments on day 15. The data are presented as the mean ± SD, *n* = 6 per group. Differences among the groups were examined with one‐way ANOVA with Tukey's posttest. ^*^ *p* < 0.05, ^**^ *p* < 0.01, ^***^ *p* < 0.001, ^****^ *p* < 0.0001, ns, not significant versus the indicated group.

Based on the excellent in vitro bioactivities of sEVs^17‐OE^ and the sustained release of sEVs mediated by GelMA hydrogel demonstrated above, we next evaluated the pro‐regenerative effects in diabetic mouse models. First, full‐skin defect wounds (diameter of ≈1.2 cm) were created on both sides of the backs of db/db mice (**Figure** **5**a). Then, a 100 µl mixture of sEVs^17‐OE^ and GelMA monomers, along with lithiumpherryl‐2′4’5‐trimethylbenzoylphosphinate (LAP), was added to the wounds and crosslinked by UV light (405 nm) for 60 s. Representative macroscopic images of full‐thickness wounds with different treatments were captured on days 0, 5, 10, and 15. As shown in Figure 5b–d, wounds treated with GelMA‐sEVs^17‐OE^ (Gel‐sEVs^17‐OE^) nearly closed and displayed fastest healing rate, compared with those treated with sEVs^17‐OE^, sEVs, GelMA hydrogel, and PBS. Specifically, on postoperative day 10, a remarkably shrunken wound was observed in the Gel‐sEV^17‐OE^ group. On day 15, a 97.24 ± 3.47% wound closure rate was achieved in Gel‐sEV^17‐OE^ group. These results were verified by H&E staining. Newly formed and neatly organized epidermis with regenerated skin appendages and complete reepithelization were observed in sEVs^17‐OE^ and Gel‐sEVs^17‐OE^ treated wounds (Figure 5e), while the re‐epithelization process was delayed in the control group. For quantitative analysis, Gel‐sEV^17‐OE^ group had the highest wound closure rate, followed by sEVs^17‐OE^, sEVs, GelMA hydrogel, and control groups (Figure 5f). The sEVs^17‐OE^‐treated groups, with or without GelMA hydrogel, had the thickest epidermis, followed by the sEVs, GelMA hydrogel, and control groups, demonstrating their efficacy in promoting re‐epithelialization (Figure 5g).

### 2.6 Gel‐sEVs^17‐OE^ Accelerated Angiogenesis and Collagen Deposition via Optimizing Cellular Functions

**Figure 6:**
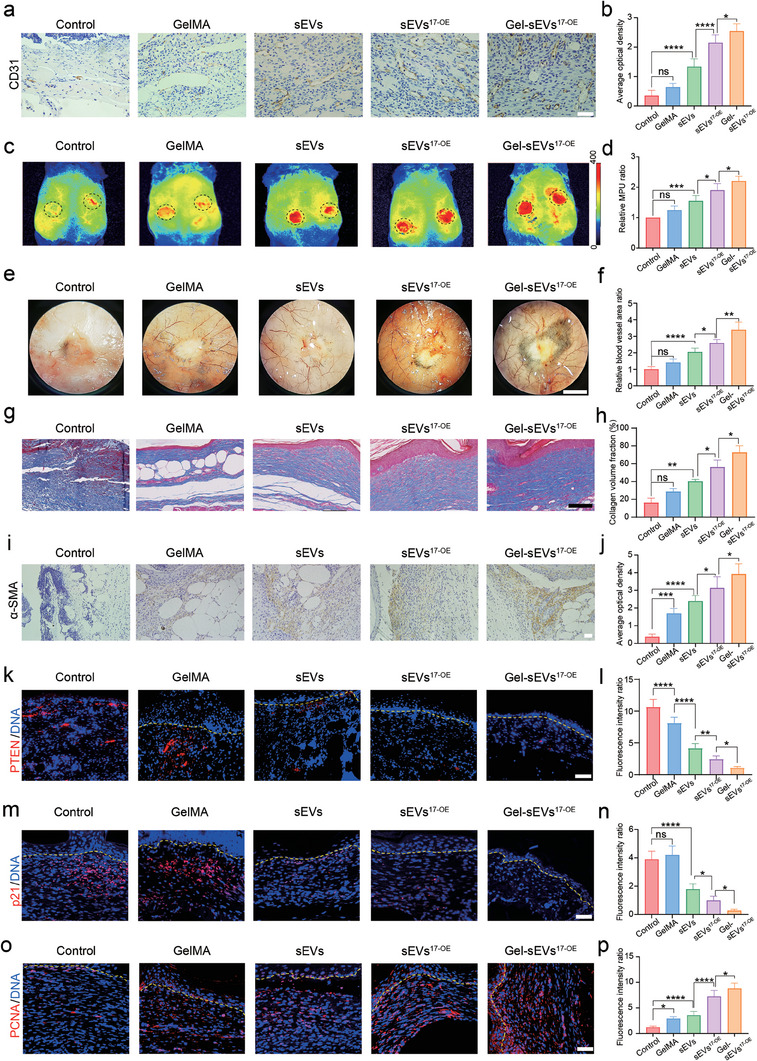
Gel‐sEVs^17‐OE^ accelerated angiogenesis and collagen deposition via alleviating senescence and enhancing proliferation. a,b) Typical immunohistochemical images and the statistical results of CD31 at wound sites on day 5 treated with PBS (Control), GelMA hydrogel, sEVs, sEVs^17‐OE^ and Gel‐sEVs^17‐OE^ to detect the vascular regeneration capacity of endothelial cells (*n* = 6 per group, scale bar = 100 µm). c) Blood flow perfusion of the wound area with the above‐mentioned treatments. d) Statistical results of (c), *n* = 6 per group. e) The new blood vessels around the wound on day 15 by the stereo microscope (scale bar = 5 mm). f) The statistical results of (e), *n* = 6 per group. g,h) Representative Masson's trichrome staining images of the wound region with the above‐mentioned treatments and quantitative analysis of collagen volume fraction on day 15 (*n* = 6 per group, scale bar = 100 µm). i,j) Typical immunohistochemical images and statistical results of α‐SMA in the wound sites on day 15 with the above‐mentioned treatments to detect the transformation of fibroblasts around the wounds (*n* = 6 per group, scale bar = 100 µm). k,l) Typical immunofluorescence images and the statistical results of PTEN in the wound sites with the treatments mentioned above on day 15 (*n* = 6 per group, scale bar = 100 µm). m,n) Representative immunofluorescence images and the statistical results of p21 in the wound sites with the treatments mentioned above on day 15 (*n* = 6 per group, scale bar = 100 µm). o,p) Representative immunofluorescence images and statistical results of PCNA at the wound sites on day 15 with the above‐mentioned treatments (*n* = 6 per group, scale bar = 100 µm). The data are presented as the mean ± SD. Differences among the groups were examined with one‐way ANOVA with Tukey's posttest. ^*^ *p* < 0.05, ^**^ *p* < 0.01, ^***^ *p* < 0.001, ^****^ *p* < 0.0001, ns, not significant versus the indicated group.

Immunohistochemical staining of CD31, a typical marker of vascular endothelial cells, was conducted on day 5 and day 10 after surgery to examine the neovascularization at wound sites (**Figure** **6**a,b, Figure S14, Supporting Information). Interestingly, on day 5, Gel‐sEVs^17‐OE^ treated wounds showed the highest expression of CD31, followed by sEVs^17‐OE^, sEVs, GelMA‐ and the control‐ treated wounds. This trend persisted for 10 days postsurgery (Figure S14, Supporting Information). To evaluate the angiogenic capacity of each group, a Laser speckle contrast imaging (LSCI) system was employed to observe blood perfusion levels around the wounds treated with the different groups on day 10 (Figure 6c). The wound treated with Gel‐sEVs^17‐OE^ clearly displayed the highest local MPU ratios (2.21 ± 0.15), indicating the most remarkable pro‐angiogenic effect, followed by the wounds treated with sEVs^17‐OE^ (1.91 ± 0.21) and sEVs (1.55 ± 0.18) (Figure 6d). Moreover, newly formed blood vessels were detected on day 15 under a stereomicroscope, as illustrated in Figure 6e. Gel‐sEVs^17‐OE^ treated wounds had the highest relative vessel area (3.40 ± 0.46), followed by sEVs^17‐OE^ (2.59 ± 0.21) and sEVs treated ones (2.05 ± 0.23) (Figure 6f). This result is consistent with those obtained from CD31 staining. Collectively, Gel‐sEVs^17‐OE^ accelerated angiogenesis at the wound site and facilitated wound healing markedly.

Collagen deposition is directly associated with HDF function during wound repair. The amount of collagen deposited was significantly higher in the sEV and sEV^17‐OE^ groups than in the control and GelMA groups (Figure 6g,h). In the Gel‐sEVs^17‐OE^ group, collagen distribution and structure were more mature, thicker, and better‐arranged (Figure 6g) on day 15. We further examined the expression level of α‐SMA, which indicated the transformation into myofibroblasts and the contraction ability of HDFs at the wound sites on day 15. As illustrated in Figure 6i,j, Gel‐sEVs^17‐OE^ group showed the highest expression level of α‐SMA, followed by sEVs^17‐OE^ and sEVs groups, indicating that many HDFs were transformed to myofibroblasts on day 15 and the healing process was significantly accelerated. After rapid closure, the remodeling phase gradually dominated over the proliferative phase. There was a significantly regressive level of α‐SMA expression in Gel‐sEVs^17‐OE^ group on day 20 (Figure S15, Supporting Information), suggesting that Gel‐sEVs^17‐OE^ administration might accelerate the healing process and deny an increased possibility of hypertrophic scars. Next, we examined the expression of p21, PTEN, and PCNA on day 15 to investigate the senescence and proliferation of skin repair cells. The control group exhibited a high level of p21 and PTEN expression, indicating that skin cells in the diabetic wounds were in a senescent state. In contrast, the expression of p21 and PTEN was decreased in sEVs, sEVs^17‐OE^, and Gel‐sEVs^17‐OE^ groups, indicating the cellular senescent state was improved. Notably, the Gel‐sEVs^17‐OE^ group had a lower level of p21 and PTEN expression than the free sEVs^17‐OE^ group (Figure 6k–n). Conversely, the highest PCNA expression was observed in the Gel‐sEVs^17‐OE^ group, indicating active proliferative behavior of skin repair cells. Therefore, Gel‐sEVs^17‐OE^ accelerated angiogenesis and collagen deposition in diabetic wounds by alleviating senescence and enhancing proliferation.

## 3 Discussion

Angiogenesis, mediated by vascular endothelial cells delivering nutrients and oxygen to the wound area, is crucial for initiating regeneration during wound healing.^[^ ^33^ ^]^ HDFs also play a crucial role as they migrate to the wound, proliferate, synthesize the extracellular matrix (ECM), and differentiate into myofibroblasts to accelerate wound contraction. However, hyperglycemia can induce cellular senescence and dysfunction, which impairs the healing capabilities of endothelial cells and HDFs, leading to slower wound repair.^[^ ^3^, ^4^, ^34^ ^]^ Based on our previous study, sEVs^17‐OE^ were fabricated and encapsulated in GelMA hydrogel for the treatment of diabetic wounds. The results showed that Gel‐sEVs^17‐OE^ effectively enhanced wound healing by optimizing the functions of endothelial cells and HDFs to promote angiogenesis and collagen deposition.

Normal skin wound healing requires the involvement of multiple cells such as vascular endothelial cells and fibroblasts. The impaired function of these cells caused by HG microenvironment is a key factor in the delayed healing of diabetic wounds. Mechanistically, the persistence of PTEN protein in diabetic wounds leads to the deactivation of the AKT/PI3K pathway, which is essential for inducing skin cell proliferation and limiting excess ROS.^[^ ^35^, ^36^ ^]^ Inhibition of PTEN not only boosts cell growth and migration but also increases the expression of Nrf2 to combat excess ROS, thus rescuing ROS‐dependent senescence in several pathological conditions.^[^ ^31^ ^]^ Additionally, p21 (a cell cycle inhibitor) has been identified as an anti‐proliferation gene,^[^ ^37^ ^]^ with its upregulation contributing to wound chronicity.^[^ ^38^ ^]^ These findings provide novel therapeutic targets for the treatment of diabetic wounds.

A group of miRNAs have been verified to regulate the expression of proteins mentioned above.^[^ ^8^ ^]^ For example, the miR‐17‐92 cluster has been reported to confer resistance to cell cycle arrest and *ras*‐induced senescence by directly silencing p21 expression in cancers.^[^ ^39^, ^40^ ^]^ Recently, we observed a protective and regenerative effect of miR‐17‐5p‐enriched sEVs on HG‐HDFs (the main cell types of dermal tissue) in vitro and in vivo, indicating that miR‐17‐5p is an efficient biomolecule for dermis regeneration.^[^ ^20^ ^]^ Meanwhile, a bunch of literature has demonstrated the beneficial role of miR‐17‐5p in other diseases, such as coronary heart disease and vascular ischemic injury by facilitating angiogenesis, osteoarthritis, renal ischemic injury, amyotrophic lateral sclerosis, and intervertebral disc degeneration by resisting degeneration and maintaining homeostasis,^[^ ^41^, ^42^, ^43^, ^44^ ^]^ highlighting its potential for treating chronic wounds.

As therapeutic molecules, miRNAs need stable and biocompatible carriers to penetrate into host cells.^[^ ^45^ ^]^ SEVs are ideal for delivering miRNAs due to their high stability, low immunogenicity, and ability to penetrate various biological barriers.^[^ ^26^ ^]^ Furthermore, sEVs secreted from MSCs display unique proregenerative effects at wound sites by regulating inflammation, boosting anagenesis, and promoting re‐epithelization via the encapsulated therapeutic RNAs such as miRNAs or long noncoding RNAs (lncRNAs).^[^ ^46^, ^47^ ^]^ However, the delivery efficacy and therapeutic outcomes of natural sEVs have proved to be unsatisfactory.

In this study, we fabricated engineered sEVs^17‐OE^ with enriched miR‐17‐5p to alleviate senescence, boost proliferation, and recover cellular function in skin repair cells in diabetic wounds. Lentiviral transfection has been widely employed to modify source cells to boost the therapeutic effects of miRNAs inside sEVs.^[^ ^48^, ^49^ ^]^ We overexpressed miR‐17‐5p inside sEVs (sEVs^17‐OE^) by genetically modifying parental cells, hucMSCs, without compromising the morphology, size distribution, expressed surface markers, and cellular internalization of sEVs. SEVs^17‐OE^ offers several advantages over other drugs and natural MSC‐sEVs developed for diabetic wounds. First, sEVs^17‐OE^ can enter target cells directly because of their phospholipid bilayer membrane structure, while the hydrophobic drugs can't enter cells directly, which limits their clinical utility. Second, sEVs^17‐OE^ is more biocompatible in comparison with other drugs. Thirdly, sEVs^17‐OE^ derived from gene transfected‐MSCs can not only deliver multiple pro‐regenerative components of MSCs‐sEVs to target cells but also amplify the therapeutic potentials of miR‐17‐5p in comparison with natural MSC‐sEVs.

Then, we investigated the effect of sEVs^17‐OE^ on the regulation of PTEN and p21‐related signaling pathways. As expected, PTEN and p21, which are negatively associated with growth and antiaging‐associated signaling pathways, were significantly upregulated upon the administration of sEVs^17‐OE^. Specifically, in vitro, sEVs^17‐OE^ repressed p21 expression and removed the inhibition on the cell cycle. Moreover, sEVs^17‐OE^ could also directly target PTEN to silence its expression, thus activating p‐AKT/AKT. This activation not only rescued ROS‐dependent senescence by downregulating p16 and p53 levels and reducing β‐galactosidase accumulation but also enhanced cell viability and migration, thus recovering the angiogenic ability of HG‐HUVECs as demonstrated by higher expression of HIF‐1α and VEGF, and facilitating collagen generation of HG‐HDFs as evidenced by the increased Col I, Col III, and α‐SMA production.

To further enhance the local retention time of sEVs^17‐OE^, we loaded sEVs^17‐OE^ into GelMA hydrogel. Various biofactors, including drugs, cells, sEVs, and autophagosomes, have been previously loaded into GelMA hydrogels to improve their potency in spinal cord injury, wound defects, spinal degeneration disorders, and cardiovascular diseases.^[^ ^24^, ^50^, ^51^, ^52^ ^]^ Generally, the porosity, mechanical strength, swelling and degradation of GelMA, and the release rate of cargo are influenced by the concentration of monomers and the substitution rate of MA.^[^ ^53^ ^]^ Accordingly, to optimize and prolong the therapeutic time of sEVs^17‐OE^, we encapsulated sEVs^17‐OE^ into GelMA hydrogel (monomer of 15%) to obtain uniformly embedded sEVs^17‐OE^ in GelMA (Gel‐sEVs^17‐OE^) after optimizing the concentration of GelMA hydrogel. The encapsulation of GelMA did not affect the morphology of the sEVs, and extended the retention time of sEVs locally. We then applied the Gel‐sEVs^17‐OE^ to treat diabetic wounds, taking PBS, GelMA hydrogel, sEVs, and sEVs^17‐OE^ as controls. We observed that Gel‐sEVs^17‐OE^ exerted growth‐promoting and anti‐senescence effects via PTEN and p21 mediated stimulation of angiogenesis and collagen accumulation, and thereby accelerated wound healing. Furthermore, we confirmed that loading sEVs^17‐OE^ into GelMA resulted in the sustained release of sEVs^17‐OE^, thus reinforcing its therapeutic potency in repairing diabetic wounds.

## 4 Conclusion

**Figure 7:**
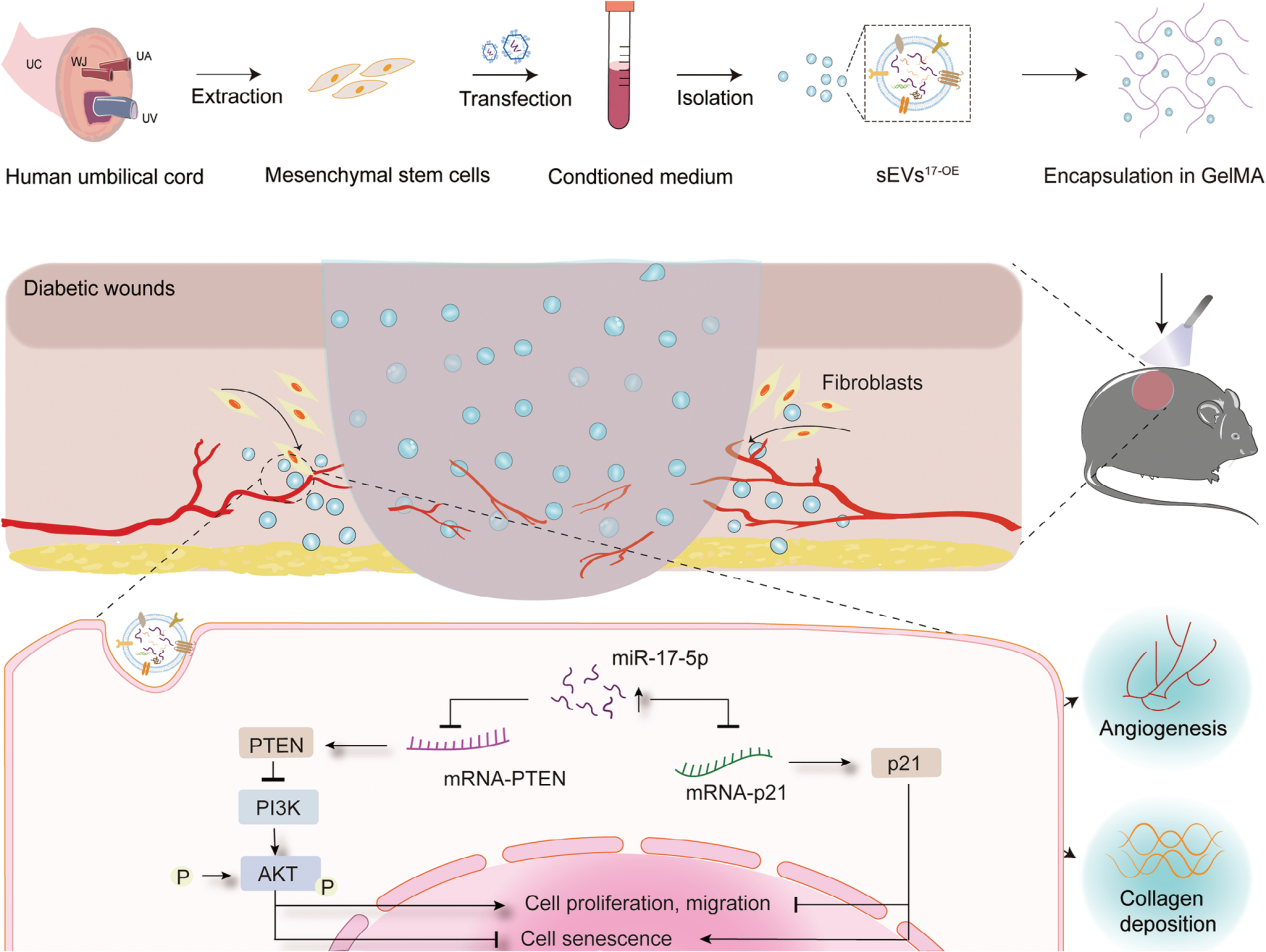
Schematic illustration showing the fabrication process of engineered hucMSC‐derived sEVs^17‐OE^‐encapsulated GelMA hydrogel and the regulation mechanisms in accelerating skin wound healing in diabetic mice via PTEN and p21‐mediated angiogenesis and collagen deposition.

As summarized in **Figure** **7**, sEVs^17‐OE^ were harvested from genetically modified hucMSCs and used to mitigate senescence and enhance the proliferation of HG‐HUVECs and HG‐HDFs by targeting the PTEN/AKT and p21 signaling pathways in vitro. In vivo, GelMA hydrogel was employed to load sEVs^17‐OE^ to achieve sustained and stable release at wound sites, improving their therapeutic potency. Administration of sEVs^17‐OE^‐loaded GelMA markedly repressed p21 expression to rescue senescence and stimulated proliferation to promote angiogenesis and collagen deposition, thus facilitating diabetic wound healing in vivo. In summary, the study shows the potential of miRNA‐engineered sEVs loaded in biomaterials as a novel strategy for therapeutic miRNA delivery to manage diabetic wounds by targeting critical cellular processes and molecular pathways.

## 5 Experiment Section

### Cell Culture

The hucMSCs were acquired, cultivated, and identified as previously reported.^[^ ^3^ ^]^ HUVECs and HDFs were obtained commercially from the Chinese Academy of Sciences’ cell bank (China) and cell passage was designated as P1 upon arrival. HUVECs and HDFs were cultured in Dulbecco's Modified Eagle's Medium (DMEM), supplemented with 10% fetal bovine serum (FBS), 1% penicillin/streptomycin, and 5 mm glucose (normal‐glucose, NG). The concentration of glucose was 35 mm for high‐glucose (HG)‐treated groups. The cells were cultured in an incubator containing 5% CO_2_ at 37 °C.

### Engineering Human Umbilical Cord Mesenchymal Stem Cells (hucMSCs) with Lentivirus Carrying miR‐17‐5p

Recombinant shuttle plasmid with oligonucleotide encoding miR‐17‐5p and packaging plasmids were purchased from Genepharma (China). These plasmids were transfected into 293T cells with Lipofectamine 3000, resulting in the generation of lentivirus carrying miR‐17‐5p (Lv‐miR‐17‐5p). A similar strategy was employed to fabricate negative control (NC) lentiviral particles (Lv‐NC). MSCs were transfected with Lv‐miR‐17‐5p or Lv‐NC to generate miR‐17‐5p‐engineered MSCs and control MSCs respectively. Followingly, puromycin (1 µg mL^−1^) was employed to select stably transfected MSCs. Positive cells were observed via the fluorescence microscope.

### Harvest and Identification of miR‐17‐5p‐Engineered MSC‐small Extracellular Vesicles (MSC‐sEVs)

SEVs from different groups were isolated as previously reported.^[^ ^3^, ^54^ ^]^ Specifically, sEVs were obtained from the supernatant by ultracentrifugation. Cell medium was collected and centrifuged at 400 g for 6 min and 2100 g for 25 min to remove the cell debris when the cell confluence reached ≈80%–90%. Next, a 0.22‐µm filter was used to filter the supernatants to remove nano‐scale non‐sEV impurities. The resulting supernatants were collected and subjected to ultracentrifugation twice (100 000 g, 75 min, 4 °C). The sEVs were then suspended in PBS for experimental use or stored at −80 °C. The size distribution and typical morphology of sEVs were conducted by nanoparticle tracking analyzer (NTA) and transmission electron microscope (TEM). Representative biomarkers for sEVs including tumor susceptibility gene 101 (TSG101), CD9, and heat shock protein 7 (HSP70) were detected by Western blot analysis.

### RNA Isolation and qPCR

Total RNAs were isolated from sEVs, sEVs^NC^, and sEVs^17‐OE^via the SeraMir Exosome RNA Purification Kit (System Biosciences, USA) according to the manufacturer's instructions. cDNAs were synthesized with the TaqMan miRNA assay kit (Applied Biosystems, USA). qRT‐PCR was performed with the ABI PRISM7900HT system with SYBR Premix ExTaqTM II (Takara Biotechnology, Japan). Stem‐loop qRT‐PCR was conducted for miRNA detection. Data were collected and relative expression levels were counted using the 2^−ΔΔCT^ method. Results were normalized to U6 small nuclear RNA for miRNAs in sEVs and cells. Primers for miRNA were synthesized by Sangon Biotech (Table S1, Supporting Information).

### DiI Staining and Cellular Internalization Assay

The DiI kit (YEASEN, China) was used to label sEVs according to the manufacturer's protocol. Briefly, sEVs were incubated with DiI dye (10 µm) solution for 20 min at room temperature (RT) in a dark environment. The stained sEVs were obtained after centrifugation at 100 000 g for 70 min and resuspended with PBS. HUVECs and HDFs were incubated with stained sEVs for 5–6 h. Subsequently, cells were fixed with 4% paraformaldehyde and washed with PBS. Then, cells were dyed with 5 µg mL^−1^ phalloidin (YEASEN, China) for 1 h and finally with Hoechst 33 342 (Beyotime, China) for 10 min (RT). Images were visualized under confocal microscopy.

### Cell Proliferation and Cell Cycle Assay

For the cell proliferation assay, Edu staining was performed using the Edu kit (BeyoClick EdU Cell Proliferation Kit with Alexa Fluor 488, Beyotime, China). HUVECs and HDFs were seeded into 24‐well plates and co‐incubated with sEVs, sEVs^NC^ or sEVs^17‐OE^ for 72 h, respectively. Again, NG‐HUVECs and NG‐HDFs were seeded into 24‐well plates and co‐incubated with PBS or the prepolymer solution of sEVs^17‐OE^‐loaded GelMA group for 72 h, respectively. Subsequently, Edu was added and co‐incubated for 3 h. The cells were fixed with 4% paraformaldehyde for 20 min, and then permeabilized with 0.3% Triton X‐100 for 15 min. Afterward, cells were incubated with Click Reaction Mixture shielded from light (RT, 30 min) and incubated with Hoechst 33342 for another 5 min.

For the cell cycle assay, HUVECs and HDFs were seeded in six‐well plates and treated with PBS, sEVs, sEVs^NC^, or sEVs^17‐OE^. The subsequent steps were performed as previously described.^[^ ^3^ ^]^ Cell cycle analysis was conducted using a cell cycle detection kit (Solarbio, China). After the treatments mentioned above, the cells were washed with PBS twice, harvested with trypsin, and then centrifuged. The cells were fixed with 70% cold alcohol overnight, followed by a PBS wash and incubation with propidium iodide for 1 h in the dark, according to the manufacturer's instructions. The distribution of cells in different phases of the cell cycle was detected using flow cytometry (BD FACS Calibur, Becton‐Dickinson, USA).

### Cell Migration Assay

The migration abilities of HG‐HUVECs and HG‐HDFs were estimated by scratch and transwell assay as previously reported.^[^ ^3^ ^]^ Briefly, cells were cultured in six‐well plates. The cells were scratched with a pipette tip (200 µL) at a 90% confluence. Then, cells were cultured at 37 °C and photographed at specific time points. The migration area was analyzed by Image J software. The migration rate (%) was measured as: (M_0_ − M_t_) / M_0_ × 100%, where M_0_ represented the initial scratch area, and M_t_ represented the residue area. For the transwell assay, cells were suspended in a serum‐free medium and cultured in the upper chamber of 24‐well plates with a polycarbonate membrane (pore diameter of 8.0 µm). Mediums containing sEVs, sEVs^NC^ or sEVs^17‐OE^ were added into the lower chamber. After 24 h, cells on the upper surface of the filter membranes were wiped off and those on the lower surface were stained with crystal violet (Solarbio, 0.1%, w/v) for 7 min. Again, mediums containing the prepolymer solution of sEVs^17‐OE^‐loaded GelMA group were added to the lower chamber for NG‐HUVECs and NG‐HDFs. The stained cells were photographed and counted under an optical microscope.

### Tube Formation Assay

Matrigel (50 µL well) was added to a pre‐cooled 96‐well plate and incubated at 37 °C for 30 min. Then, HUVECs (2 × 10^4^ cells per well) from different groups (NG, HG, HG + sEVs, HG + sEVs^NC^, HG + sEVs^17‐OE^) were added to the Matrigel‐coated wells. After incubating for 4–6 h, tube‐like structures formed on the Matrigel were photographed via an optical microscope. Next, HUVECs (2 × 10^4^ cells per well) from the NG group and the prepolymer solution of sEVs^17‐OE^‐loaded GelMA group were added to the Matrigel‐coated wells, respectively. Tube formation was photographed after incubation. Data were analyzed by Image J.

### Quantification of Intracellular ROS

The concentration of ROS in HDFs and HUVECs was estimated by an oxidation‐sensitive fluorescent probe dye, 2′,7′‐dichlorodihydrofluorescein diacetate (DCFH‐DA) and hydroethidine (Beyotime, China). The cells were seeded in a 24‐well plate and the next day were incubated with an equal dose (20 µL) of different types of sEVs or PBS for 24 h, followed by washing with PBS. Then, the cells were incubated with DCFH‐DA (10 µm) for 20 min and Hoechst 33 342 for 10 min. ROS level inside the cells was evaluated by flow cytometry, and intracellular fluorescence was visualized by a confocal microscopy.

### Western Blot Analysis

The samples including sEVs, HUVECs, and HDFs were homogenized in lysis buffer containing proteinase and phosphate kinase inhibitors (Solarbio, China). Proteins (20 µg) were mixed with loading buffer, electrophoresed via SDS‒PAGE gels, and then transferred to polyvinylidene fluoride (PVDF) membranes (Millipore, USA). After blocking in 5% nonfat milk, membranes were co‐incubated with primary antibodies against tumor susceptibility gene 101 (TSG101), heat shock protein (HSP70), CD9, GAPDH, Calnexin, PTEN, phospho‐ protein kinase B (p‐AKT), AKT, hypoxia‐inducible factor – 1α (HIF‐1α), vascular endothelial growth factor (VEGF), p21, p16, p53, Type I collagen (Col I), Type III collagen (Col III), α‐Smooth Muscle Actin (α‐SMA), and β‐actin (1:1000, CST, USA) at 4 °C overnight. Afterward, membranes were washed with Tris‐Buffered SalineTween‐20 (TBST) and co‐incubated with horseradish peroxidase‐conjugated anti‐mouse or anti‐rabbit IgG (1:5000, ZSGB‐BIO, China) for 1 h (RT). Bands were visualized by an enhanced chemiluminescence  kit (Solarbio, China). The gray value assay representing the protein expression level was quantified by Image J.

### Preparation and Characterization of GelMA Hydrogel and Gel‐sEVs

GelMA monomer and lithiumpherryl‐2,4,5‐trimethylbenzoylphosphinate (LAP) were bought from Engineering for Life (China) and GelMA hydrogel was prepared as previously reported. First, GelMA monomer with a concentration of 5%, 10%, and 15% was mixed with LAP initiator (0.25%, w/v). Next, the solutions were filter‐sterilized via a 0.22 µm filter and crosslinked by exposure to 405 nm light for 1 min. Each sample was stored at −80 °C and then freeze‐dried overnight. The samples were placed on the conducive tape and treated with gold sputtering for 1 min. Then they were observed and photographed with a scanning electron microscope (SEM, ZEISS Gemini).

### Swelling Ratio and Mechanical Properties of GelMA Hydrogel

The swelling ratio of GelMA hydrogel was estimated by using a weight‐based method as previously reported.^[^ ^24^ ^]^ Briefly, the cross‐linked cylindrical specimens (with a diameter of 10 mm and a height of 5 mm) were primarily weighed as W_0_. Then, specimens were fully immersed in PBS and incubated for the designed time. After removing water from the surface, the specimens were weighed at various time points as Wt. The swelling ratio (%) was evaluated as: (W_t_–W_0_) / W_0_ × 100%. To evaluate the mechanical properties of GelMA hydrogel samples (with a length of 40 mm and width of 10 mm), they were clamped at one end and pulled at the other end at a speed of 2 mm min^−1^ by using an electric universal test machine (CMT6103, MTS).

### Tissue Adhesiveness Assays

Ex‐vivo adhesion assays were conducted using pig skins to examine the adhesiveness of the Gel‐sEVs^17‐OE^ as literature reported previously.^[^ ^55^, ^56^, ^57^ ^]^ Briefly, the fresh pig skins were cut into strips (50 × 15 mm) before being washed with 1 m sodium hydroxide (3 times) and deionized water to get rid of adipose tissue. Subsequently, the mixture of GelMA prepolymer, sEVs^17‐OE^, and red edible pigment (Sugarman, China) was applied onto the surface of the porcine and achieved in situ gelation after UV curing to test the adhesiveness by deforming the porcine skins to simulate dynamic and humid environments. Then, tissue adhesion of Gel‐sEVs^17‐OE^ was conducted by lap shear test. A 20wt% gelatin solution was spread onto the surface of the glass to mimic the surface of porcine skin. The mixture of GelMA prepolymer, sEVs^17‐OE^, and red edible pigment was applied on the two independent stimulated skin and then they were integrated closely under a certain amount of pressure with a contact area of 2 × 2 cm as illustrated (Figure S9, Supporting Information). Shear adhesive forces were measured using 100 g weights.

### Examination of Degradation Behavior of GelMA Hydrogel

The degradation behavior assay was performed according to the previous report.^[^ ^24^ ^]^ Briefly, the cross‐linked cylindrical specimens with a 10 mm diameter and a 5 mm height were soaked in sterile PBS solution at 37 °C continuously for weeks. The fluid on the surface of the samples was removed, and the samples were weighed at predetermined time points. The mass remaining as a percentage was calculated as W_t_  W_0_ × 100%. The samples were originally weighed as W_0_ and reweighed as W_t_ at pre‐arranged time points.

### Analysis of Sustained Release of sEVs from Gel‐sEVs in Vitro

The DiI‐stained sEVs^17‐OE^ were mixed with GelMA monomer and LAP, and then cross‐linked under ultraviolet radiation for 1 min to obtain the Gel‐sEVs^17‐OE^. Next, Gel‐sEVs^17‐OE^ was embedded in a 24‐well plate containing PBS and the supernatant was harvested every other day for a total of 20 days. The fluorescence intensity of released sEVs^17‐OE^ in the supernatant at 570 nm was measured by using a microplate reader. In vitro, the internalization of DiI‐stained sEVs^17‐OE^ released from 15% GelMA hydrogel by target cells was examined and photographed by confocal microscopy.

### Live/Dead Staining Assay

To evaluate the biocompatibility of sEV^17‐OE^‐loaded GelMA group and GelMA group, a live/dead staining assay was performed via a staining kit (Solarbio, China). Briefly, HG‐HUVECs and HG‐HDFs were seeded into 24‐well plates with a density of 20 000 cells per well and incubated with the prepolymer solution of GelMA, sEV^17‐OE^‐loaded GelMA group, and the normal saline (NS) as control. After 72 h coincubation, cells were washed with PBS and stained subsequently with Calcein‐AM mixture (green fluorescent dye), propidium iodide (red fluorescent dye) and 4′6‐diamidino‐2‐phenylindole (DAPI). Stained cells were examined and photographed using a CLSM.

### Animal Experiment

The animal experiment was performed under strict supervision and approved by the Animal Research Committee of PLA General Hospital in Beijing. Eight‐week‐old male diabetic mice (BKS‐Dock Leprem2Cd479, DB/db) were bought from the Jicui Co. (China) and raised in a standard experiment animal feeding environment. After anesthetized with 4% chloral hydrate, full‐thickness dorsal skin wounds were created on both sides of the mice. And then these mice were randomly divided into five groups including 1) PBS, 2) GelMA, 3) sEVs, 4) sEVs^17‐OE^, and 5) Gel‐sEVs^17‐OE^. For groups (3) and (4), 100 µL of sEVs or sEVs^17‐OE^ (2 × 10^10^ particles mL^−1^) solution was injected surrounding the wounds at four spots (25 µL per site), respectively. For groups (2) and (5), 100 µL of GelMA or Gel‐sEVs^17‐OE^ solution was dropped on the wounds and then cross‐linked under ultraviolet radiation for at least 1 min on the day of surgery. The wounds in each group were imaged and examined with a measuring tape and the wound tissues were harvested on days 0, 5, 10, and 15 after operation. Wound areas were estimated by Image J software. The wound closure rate was calculated as: (A_0_–A_t_) / A_0_ × 100%, where A_0_ referred to the primary wound area and A_t_ referred to the wound area at specific time points. For sustained release of sEVs from Gel‐sEVs in vivo, the fluorescence intensity of DiI‐stained sEVs^17‐OE^ was examined by using PerkinElmer IVIS Spectrum on days 1, 2, 3, and 5. To observe the formation of new blood vessels, the underside of the skin was photographed under a stereomicroscope (Leica Germany) on day 15. The ratio of the neovessel area to the detected area was calculated. The relative blood vessel area rate of each group was compared with the control group.

### Histological Analysis and Immunohistochemistry

At the designed time points, wound tissues were collected and fixed with paraformaldehyde. After being dehydrated and embedded in paraffin, the tissues were cut into 5 µm‐thick sections. Then, they were dyed with hematoxylin and eosin (H&E) and Masson's trichrome, respectively. The measurement of wound width and collagen volume fraction was quantified as previously reported.^[^ ^3^ ^]^ Immunohistochemistry staining of CD31 (Abcam, UK, 1:100) and α‐SMA (CST, USA, 1:800) was performed to label endothelial cells and myofibroblasts. Immunofluorescence staining of p21 (CST, USA, 1:400), PCNA (Abcam, UK, 1:1000), and PTEN (CST, USA, 1:200) were performed to demonstrate the cellular senescence and proliferative ability at wound sites. The time points of tissue harvest for CD31 staining were day 5 and day 10. The time point of tissue harvest for collagen deposition, PTEN staining, p21 staining, and PCNA staining was day 15. The time points of tissue harvest for α‐SMA staining were day 15 and day 20.

### Blood Perfusion Evaluation in Vivo

Laser speckle contrast imaging (LSCI) was employed to investigate the blood perfusion around the wounds on day 15, following established protocols.^[^ ^3^ ^]^ Images were photographed with the same scan site dimension at a fixed distance from the wound. PIMSoft (Moor Instruments Ltd, UK) was used to take flux pictures for each wound, measuring the mean perfusion units (MPUs) by comparing MPUs at the wound sites with those around the wounds.

### Biocompatibility Studies (in Vivo)

For in vivo biocompatibility evaluation of GelMA hydrogel and Gel‐sEVs^17‐OE^, vital organs of mice, including heart, lung, liver, kidney, and spleen, were harvested after 15 days of topical administration of PBS (control), GelMA hydrogel, and Gel‐sEVs^17‐OE^ and fixed in paraformaldehyde. The tissues were cut into 5 µm‐thick sections after dehydration and embedment in paraffin. Then, sections were stained with H&E respectively and assessed as described previously.^[^ ^58^ ^]^

### Cell Counting Kit‐8 (CCK‐8) Assay

HG‐HUVECs and HG‐HDFs were respectively seeded into 96‐well plates at 2 × 10^3^ cells per well and co‐incubated with the designed concentration of sEVs^17‐OE^ of 10 µL per well for 12–24 h. After the co‐incubation, 10 µL CCK‐8 reagent (Solarbio, China) was added to each well for 2 h incubation under 37 °C. The 450 nm absorbance value was detected by a microplate reader to assess the cell viability and each group was conducted in triplicate.

### Statistical Analysis

Data were reported as mean ± standard deviation (SD) from at least three independent experiments. Statistical variances between two independent groups were analyzed using an unpaired t‐test via GraphPad Prism Software 9.5. The analysis of three or more groups was examined using one‐way ANOVAs with Tukey's posttest to examine statistical significance among different groups. *p* < 0.05 was considered to be the cut‐off value of statistical significance. The specific sample sizes (n) for each statistical analysis were displayed in figure legends. Notes for statistical significance in figures were as follows: ns represents not significant, * represents *p* < 0.05, ** represents *p* < 0.01, *** represents *p* < 0.001, **** represents *p* < 0.0001, and # represents *p* < 0.05.

### Ethics Statement

All practices on animals were performed in line with the National Institutes of Health Guide for the Care and Use of Laboratory Animals and approved by the Animal Research Committee of PLA General Hospital in Beijing, China.

## Conflict of Interest

The authors declare no conflict of interest.

## Author Contributions

Q.W., J.S., and S.M. contributed equally to this work and should be considered co‐first authors. Q.W., J.S., and S.M. designed the experiments, performed the experiments, analyzed the data, and wrote and edited the manuscript. Y.W., K.M., and B.L. performed experiments, analyzed data, and edited manuscripts. Z.C., Q.H., and W.H. performed the experiments and analyzed the data. Z.W. and L.T. performed the experiments and edited the manuscript. X.L. conceptualized and funded the work, designed the experiments, and revised the manuscript. T.L. conceptualized, designed the experiments revised the manuscript and supervised the work. X.F. and C.Z. conceptualized and funded the work, designed the experiments, revised the manuscript, and supervised the work.

## Supporting information

Supporting Information

## Data Availability

The data that support the findings of this study are available from the corresponding author upon reasonable request.
