# Supplementary material for: MiR‐17‐5p‐engineered sEVs Encapsulated in GelMA Hydrogel Facilitated Diabetic Wound Healing by Targeting PTEN and p21
Source: Adv Sci (Weinh). 2024 Jan 29;11(13):2307761. doi: 10.1002/advs.202307761 (PMC10987139; doi:10.1002/advs.202307761)
Supplement: Supplementary file 1 — Supporting Information [file ADVS-11-2307761-s001.pdf]

ADVANCED SCIENCE
Open Access

# Supporting Information

for *Adv. Sci.*, DOI 10.1002/advs.202307761

MiR-17-5p-engineered sEVs Encapsulated in GelMA Hydrogel Facilitated Diabetic Wound Healing by Targeting PTEN and p21

*Qian Wei, Jianlong Su, Sheng Meng, Yaxi Wang, Kui Ma, Bingmin Li, Ziqiang Chu, Qilin Huang, Wenzhi Hu, Zihao Wang, Lige Tian, Xi Liu*, Tanshi Li*, Xiaobing Fu* and Cuiping Zhang**

# Supporting Information

# MiR-17-5p-engineered sEVs encapsulated in GelMA hydrogel facilitated diabetic wound healing by targeting PTEN and p21 to enhance the regenerative ability of endothelial cells and fibroblasts

*Qian Wei, Jianlong Su, Sheng Meng, Yaxi Wang, Kui Ma, Bingmin Li, Ziqiang Chu, Qilin Huang, Wenzhi Hu, Zihao Wang, Lige Tian, Xi Liu,* Tanshi Li,* Xiaobing Fu,* and Cuiping Zhang**

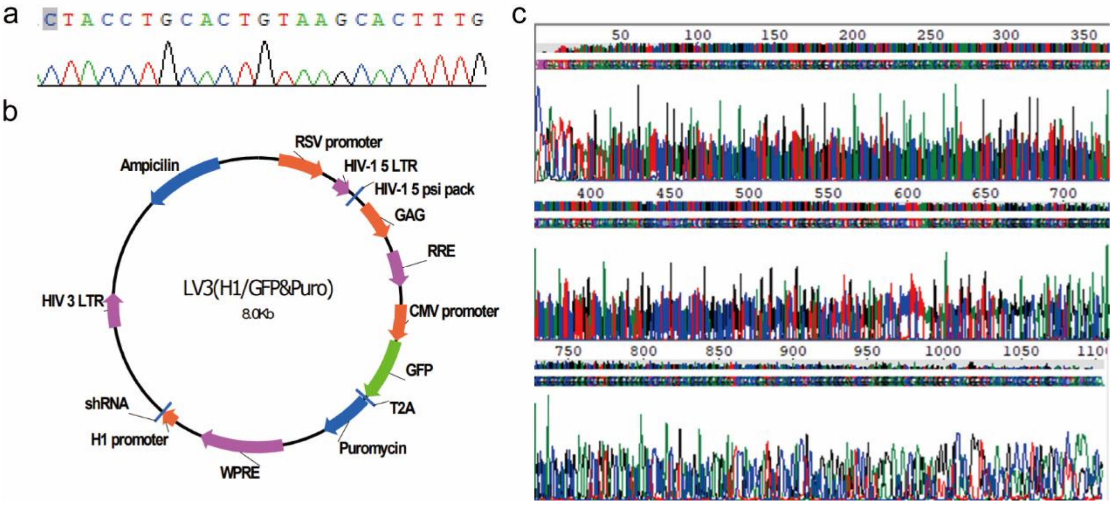


**Figure S1.** Construction and sequencing analysis of lentivirus plasmid containing miR-17-5p. a) The reverse sequencing result. b) Skeleton pattern diagram of lentivirus vector. c) Sequencing results of the vector containing the fully encoded sequence of miR-17-5p.

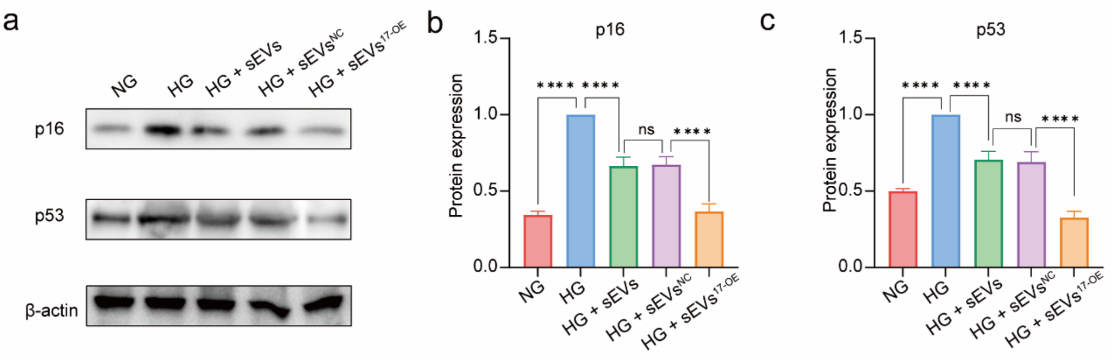


**Figure S2.** SEVs$^{17\text{-OE}}$ downregulated expression levels of p16 and p53 in HG-HUVECs. a) Represented images of western blot analysis showing the protein levels of p16 and p53 in HUVECs treated with NG, HG, HG + sEVs, HG + sEVs$^{NC}$ and HG + sEVs$^{17\text{-OE}}$. b-c) Quantification of protein levels in different groups mentioned above. Protein expression was normalized to β-actin levels (n = 3 per group). The data are presented as the mean ± SD. Differences among the groups were examined with one-way ANOVA with Tukey's posttest. ****$P < 0.0001$, ns, not significant versus the indicated group.

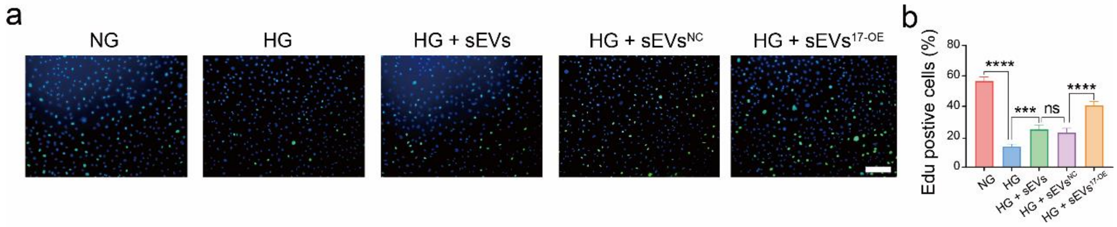


**Figure S3.** SEVs$^{17\text{-OE}}$ recovered proliferation abilities of HG-HUVECs. a) Immunofluorescence images of the Edu (green) assay on HUVECs treated with NG, HG, HG + sEVs, HG + sEVs$^{NC}$ and HG + sEVs$^{17\text{-OE}}$ (scale bar = 100 μm). The cell nuclei were dyed blue with DAPI. b) Quantification of the percentage of Edu-positive HUVECs from different treatments (n = 3 per group). The data are presented as the mean ± SD. Differences among the groups were examined with one-way ANOVA with Tukey's posttest. ***$P < 0.001$, ****$P < 0.0001$, ns, not significant versus the indicated group.

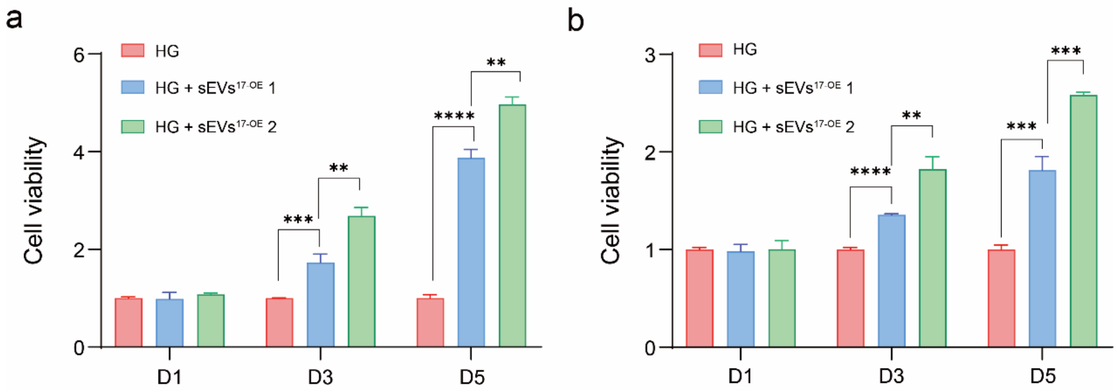


**Figure S4.** SEVs$^{17\text{-OE}}$ recovered proliferation abilities of HG-HUVECs (a) and HG-HDFs (b) in a dose-dependent manner. sEVs$^{17\text{-OE}}$ 1 and sEVs$^{17\text{-OE}}$ 2 refer to sEVs with concentration of $1 \times 10^{10}$ particles ml$^{-1}$ and $2 \times 10^{10}$ particles ml$^{-1}$, respectively. The data are presented as the mean ± SD (n = 3 per group). Differences among the groups were examined with one-way ANOVA with Tukey's posttest. ***P* < 0.01, ****P* < 0.001, *****P* < 0.0001 versus the indicated group.

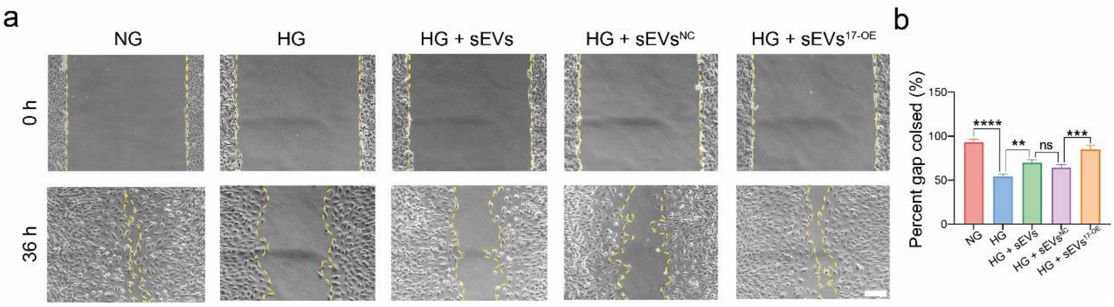


**Figure S5.** SEVs$^{17\text{-OE}}$ recovered migration abilities of HG-HUVECs. a) Representative images of scratch assay reflecting migration abilities of HUVECs with different treatments (NG, HG, HG + sEVs, HG + sEVs$^{NC}$ and HG + sEVs$^{17\text{-OE}}$). Yellow dotted lines marked the scratch edges (scale bar = 100 μm). b) Histogram showing the percentage of the closed area covered by migrated HUVECs from different groups to the original scratch area. The data are presented as the mean ± SD (n = 3 per group). Differences among the groups were examined with one-way ANOVA with Tukey's posttest. **$P$ < 0.01, ***$P$ < 0.001, ****$P$ < 0.0001, ns, not significant versus the indicated group.

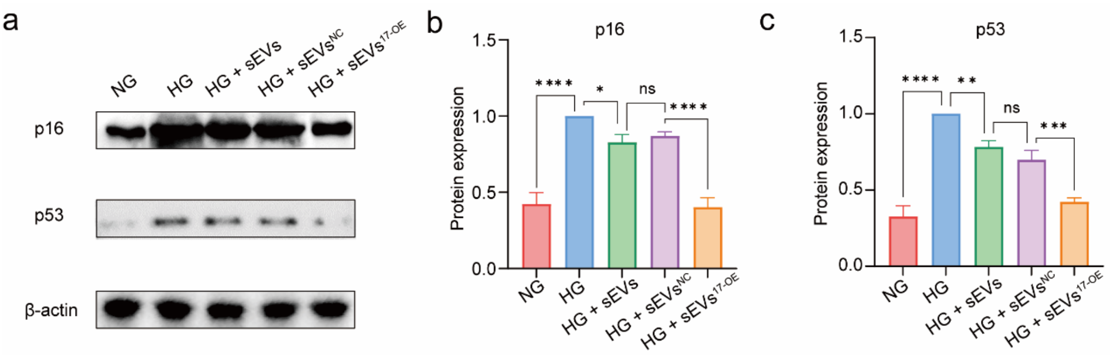


**Figure S6.** SEVs$^{17\text{-OE}}$ downregulated expression level of p16 and p53 in HG-HDFs. a) Represented images of western blot analysis showing the protein levels of p16 and p53 in HDFs treated with NG, HG, HG + sEVs, HG + sEVs$^{NC}$ and HG + sEVs$^{17\text{-OE}}$. b-c) Quantification of protein levels in different groups above. Protein expression levels were normalized to β-actin levels. The data are presented as the mean ± SD (n = 3 per group). Differences among the groups were examined with one-way ANOVA with Tukey's posttest. *$P$ < 0.05, **$P$ < 0.01, ***$P$ < 0.001, ****$P$ < 0.0001, ns, not significant versus the indicated group.

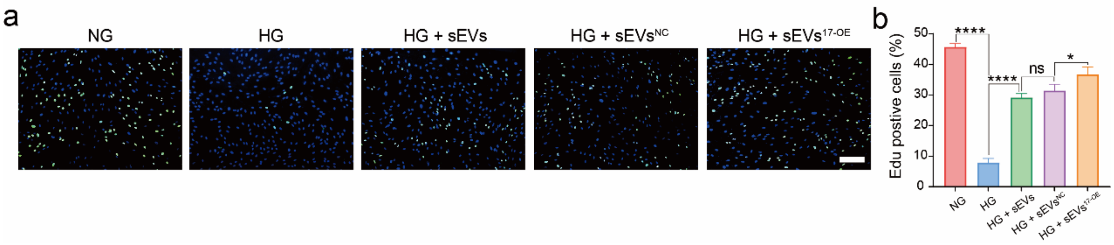


**Figure S7.** SEVs$^{17\text{-OE}}$ recovered cell viability of HG-HDFs. a) Immunofluorescence imaging of Edu (green) incorporation into proliferating cells (scale bar = 100 μm). The cell nuclei were dyed blue with DAPI. b) Quantification of the percentage of Edu-positive HDFs from different treatments. The data are presented as the mean ± SD (n = 3 per group). Differences among the groups were examined with one-way ANOVA with Tukey's posttest. *$P$ < 0.05, ****$P$ < 0.0001, ns, not significant versus the indicated group.

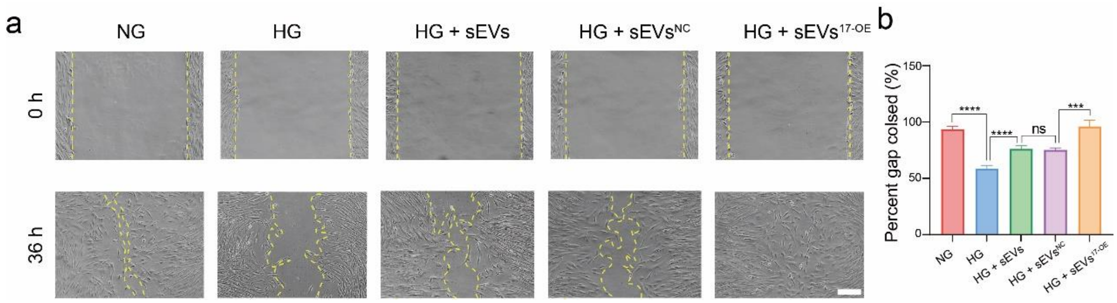


**Figure S8.** SEVs$^{17\text{-}OE}$ restored migration ability of HG-HDFs. a) Representative images of scratch assay showing the migration ability of HDFs treated with NG, HG, HG + sEVs, HG + sEVs$^{NC}$ and HG + sEVs$^{17\text{-}OE}$. Yellow dotted lines marked the current scratch edges (scale bar = 100 μm). b) Histogram showing the percentage of the closed area covered by migrated HDFs from above groups to the original scratch area. The data are presented as the mean ± SD (n = 3 per group). Differences among the groups were examined with one-way ANOVA with Tukey's posttest. ***$P < 0.001$, ****$P < 0.0001$, ns, not significant versus the indicated group.

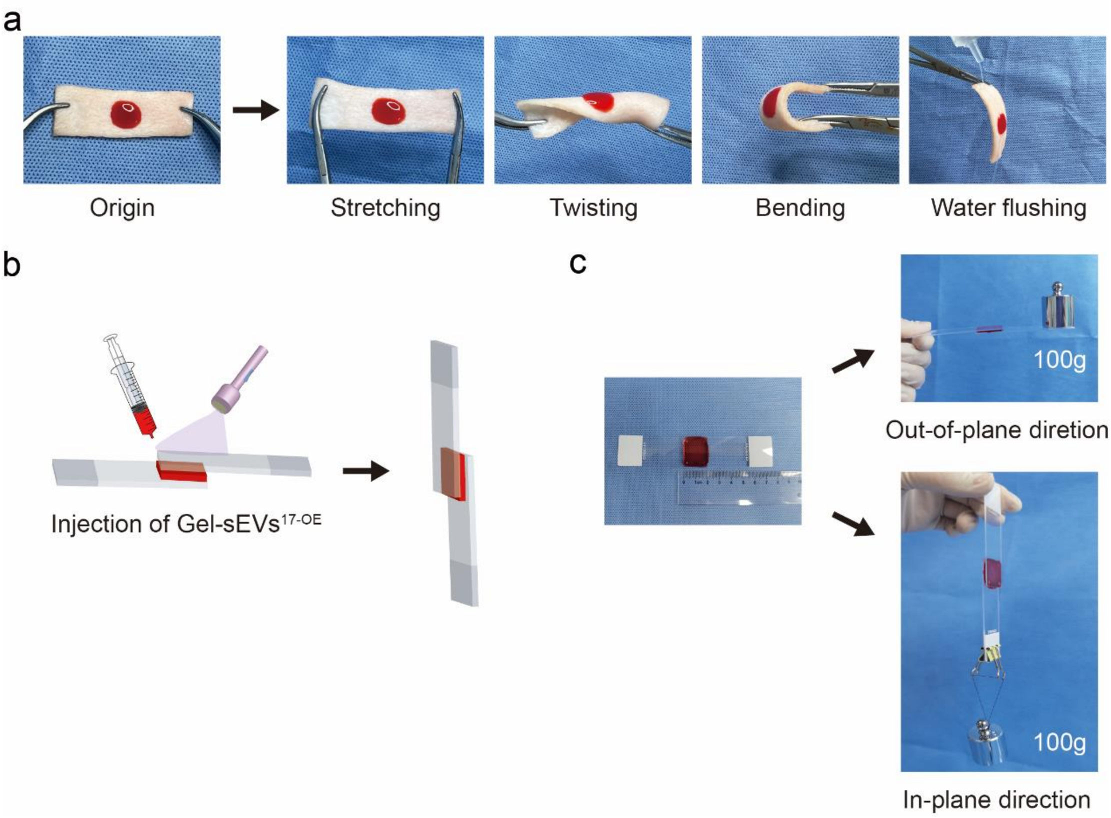


**Figure S9.** Tissue adhesiveness of Gel-sEVs$^{17\text{-OE}}$. a) Images showing red pigment-labelled Gel-sEVs$^{17\text{-OE}}$ -adhered pig skin undergoing repetitive dynamic deformation including stretching, twisting, bending and water flushing *in situ*. b) Diagrammatic sketch of adhesion test to examine the shear strength of Gel-sEVs$^{17\text{-OE}}$. c) Two pieces of 20% gelatin-coated glass slides with their overlapping region (2 cm × 2 cm) bonded together with the Gel-sEVs$^{17\text{-OE}}$ could withstand a weight of 100 g both in out-of-plane and in-plane directions.

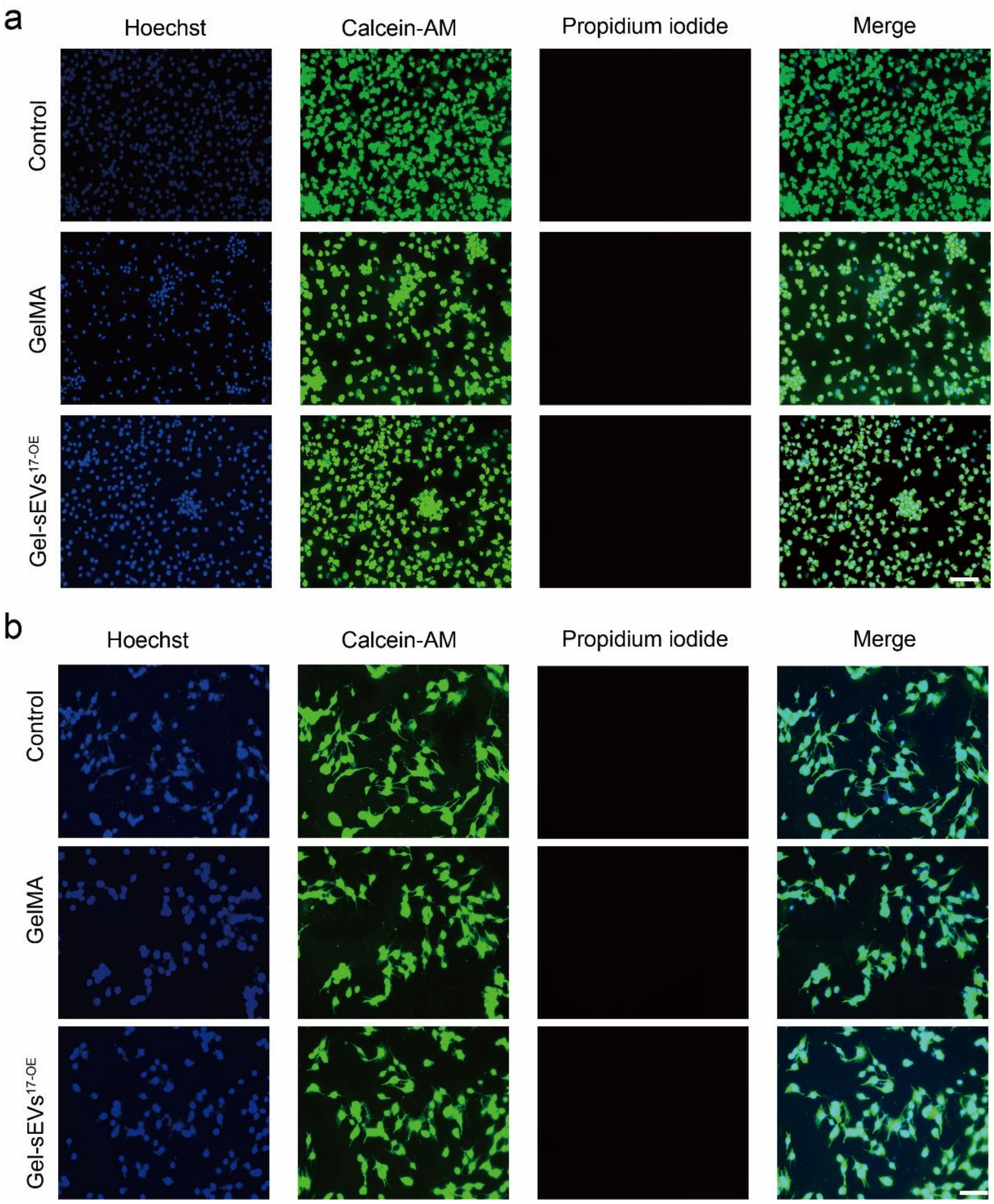


**Figure S10.** Biocompatibility evaluation of GelMA and Gel-sEVs$^{17\text{-OE}}$ *in vitro*. a) Representative images of live/dead staining assay performed on the HG-HUVECs from control group, GelMA group and Gel-sEVs$^{17\text{-OE}}$ group (n = 3 per group, scale bar = 200 μm). b) Images of live/dead staining assay conducted on the HG-HDFs from perspective groups (n = 3 per group, scale bar = 100 μm).

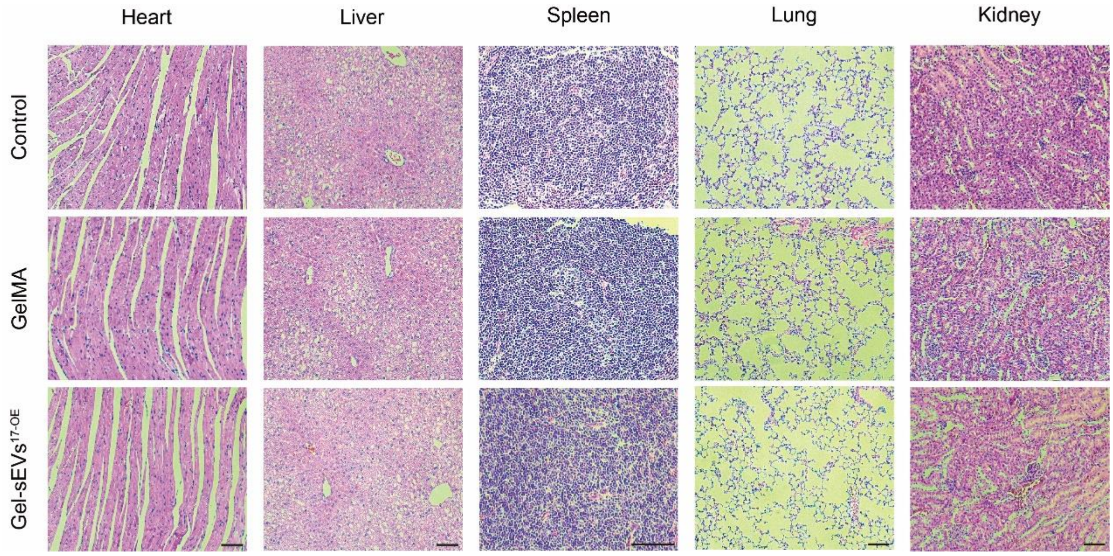


**Figure S11.** Biocompatibility evaluation of GelMA hydrogel and Gels-sEVs$^{17\text{-OE}}$ *in vivo*. H&E staining of heart, liver, spleen, lung, and kidney of the mice treated with GelMA hydrogel and Gels-sEVs$^{17\text{-OE}}$ for 15 days demonstrated no histological abnormalities or immune cell infiltration (scale bar = 100 μm).

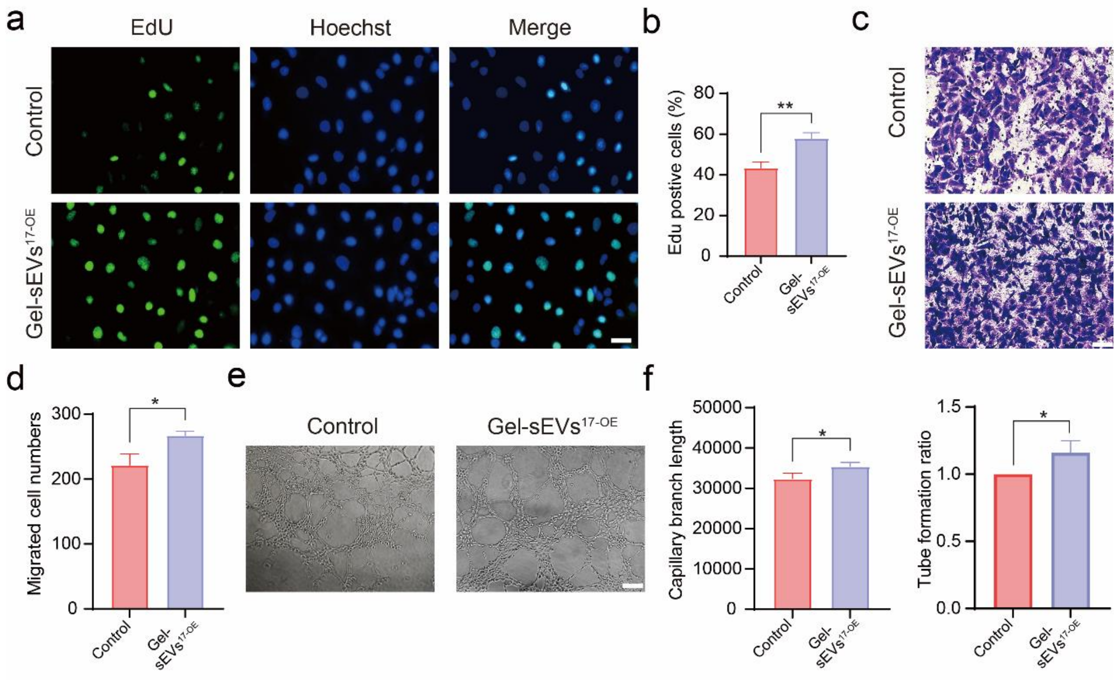


**Figure S12.** Gel-sEVs$^{17\text{-OE}}$ enhanced function of NG-HUVECs. a) Immunofluorescence imaging of Edu (green) incorporation into proliferating cells (scale bar = 50 μm). DAPI represents the nucleus in blue. b) Quantification of the percentage of Edu-positive NG-HUVECs from different treatments (n = 3 per group). c-d) Transwell assay representing migration properties of NG-HUVECs with above treatments (n = 3 per group, scale bar = 100 μm). e-f) Representative images of *in-vitro* angiogenesis assay (scale bar = 100 μm), quantification results of capillary branch length, and tube formation ratio in NG-HUVECs treated with different groups (n = 3 per group). Differences among the groups were examined with one-way ANOVA with Tukey's posttest. *$P < 0.05$, **$P < 0.01$, *ns*, not significant versus the indicated group.

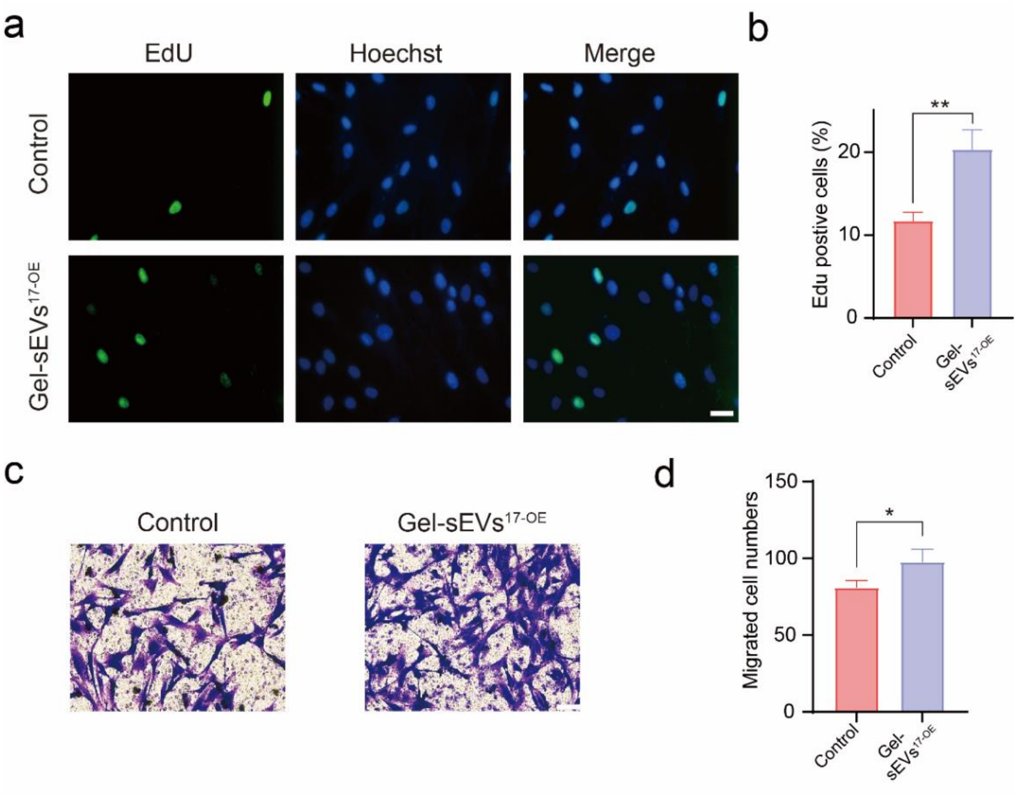


**Figure S13.** Gel-sEVs$^{17\text{-}OE}$ enhanced function of NG-HDFs. a) Immunofluorescence imaging of Edu (green) incorporation into proliferating cells (scale bar = 50 μm). DAPI represents the nucleus in blue. b) Quantification of the percentage of Edu-positive NG-HDFs from different treatments (n = 3 per group). c-d) Transwell assay representing migration properties of NG-HDFs with above treatments (n = 3 per group, scale bar = 100 μm). Differences among the groups were examined with one-way ANOVA with Tukey's posttest. *$P < 0.05$, **$P < 0.01$, *ns*, not significant versus the indicated group.

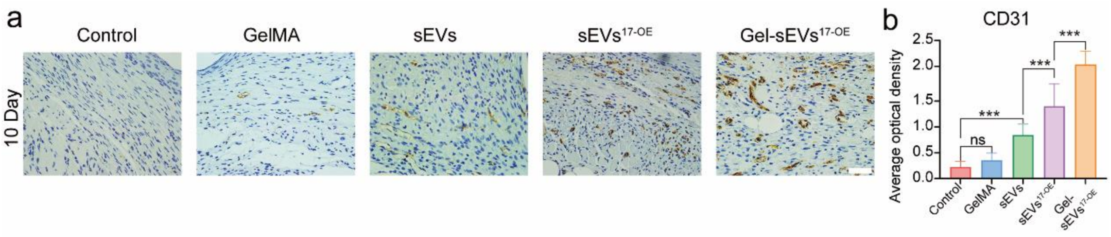


**Figure S14.** Gel-sEVs$^{17\text{-OE}}$ promoted angiogenesis at wound sites. a) Typical immunohistochemical images of CD31 on day 10 reflecting angiogenesis in wounds treated with PBS, GelMA, sEVs, sEVs$^{17\text{-OE}}$ and Gel-sEVs$^{17\text{-OE}}$ (scale bar = 100 μm). b) Statistical results of a. The data are presented as the mean ± SD (n = 6 per group). Differences among the groups were examined with one-way ANOVA with Tukey's posttest. ****P* < 0.001, ns, not significant versus the indicated group.

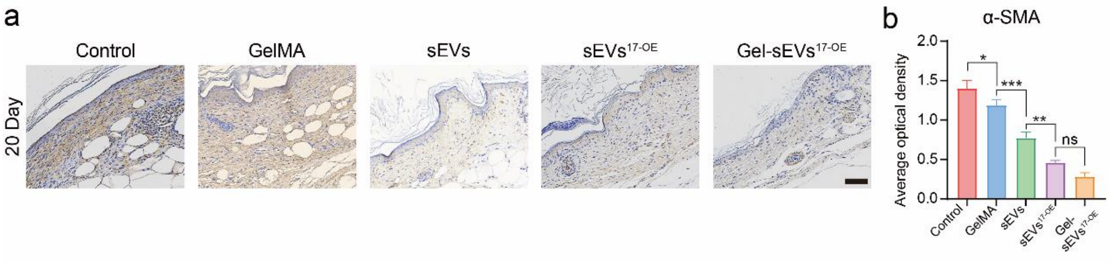


**Figure S15.** Expression of α-SMA at wound sites on day 20. a) Typical immunohistochemical images of α-SMA on day 20 at wound sites treated with PBS, GelMA, sEVs, sEVs$^{17\text{-OE}}$ and Gel-sEVs$^{17\text{-OE}}$ (scale bar = 200 μm). b) Statistical results of (a). The data are presented as the mean ± SD (n = 6 per group). Differences among the groups were examined with one-way ANOVA with Tukey's posttest. *$P < 0.05$, **$P < 0.01$, ***$P < 0.001$, ns, not significant versus the indicated group.

**Table S1. Sequence of specific primers used for qPCR analysis.**

| Gene name | Primer sequence (5′ to 3′) |
|---|---|
| GAPDH | F: AGAAGGCTGGGGCTCATTTG<br>R: GCAGGAGGCATTGCTGATGAT |
| U6 | F: CTCGCTTCGGCAGCACA<br>R: AACGCTTCACGAATTTGCGT<br>RT: GTCGTATCCAGTGCAGGGTCCGAGGTATTCGCACTGGATACGACAAAATATG |
| Universal | GTGCAGGGTCCGAGGT |
| miR-17-5p | F: GCGCAAAGTGCTTACAGTGC<br>RT: GTCGTATCCAGTGCAGGGTCCGAGGTATTCGCACTGGATACGACCTACCT |
